# A New Alkylation of Aryl Alcohols by Boron Trifluoride Etherate

**DOI:** 10.3390/molecules24203720

**Published:** 2019-10-16

**Authors:** Ndze Denis Jumbam, Yamkela Maganga, Wayiza Masamba, Nomthandazo I. Mbunye, Esethu Mgoqi, Sphumusa Mtwa

**Affiliations:** Department of Chemical and Physical Sciences, Faculty of Natural Sciences, Walter Sisulu University, Nelson Mandela Drive, Mthatha 5117, South Africa; njumbam@wsu.ac.za (N.D.J.); magangayamkela@gmail.com (Y.M.); mbunyenomthandazo@gmail.com (N.I.M.); esemgoqi12@gmail.com (E.M.); mtwasphumusa@gmail.com (S.M.)

**Keywords:** aryl alcohols, aryl ethyl ethers, boron trifluoride etherate, ethylation, functional groups

## Abstract

The ethylation of aryl alcohols by an ethyl moiety of boron trifluoride etherate is described. The reaction proceeded cleanly and afforded good yields of the corresponding aryl ethyl ethers. It tolerated the presence of functional groups such as aryl, alkyl, halogens, nitro, nitrile, and amino. However, the presence of amino or nitro groups ortho to a hydroxyl group of an aryl compound drastically reduced the yields of the anticipated products due to the chelation of the aforementioned functional groups with boron trifluoride etherate. A nitrogen atom in the aromatic ring system, as exemplified by hydroxypyridine and 8-hydroxyquinoline, completely inhibited the reaction. Resorcinol, hydroquinone, and aryl alcohols with aldehyde functions decomposed under the reaction conditions.

## 1. Introduction

The unprecedented ethylation reaction of carboxylic acids by an ethyl moiety of boron trifluoride etherate (BTE) to form corresponding ethyl esters in the absence of ethyl alcohol may have opened up a new synthetic route for the formation of aryl ethyl ethers, thanks to the observed simultaneous formation of ethyl *p*-ethoxybenzoate from *p*-hydroxybenzoic acid [1]. 

Aryl ethyl ethers find varied applications in industry ranging from solvents, paints, cosmetics, and pharmaceuticals [2]. For example, *p*-ethoxyaniline is the precursor for the production of the analgesic phenacetin [3,4], β-ethoxynaphthalene (also known as nerolin) is used as a fixative in perfumery [5] while some derivatives of aryl ethers exhibit antioxidative properties [6]. In light of the aforementioned properties of aryl ethyl ethers and their potential applications in the chemical and pharmaceutical industries, we set out to explore the alkylation of various phenols using this new ethylating agent. 

**Figure 1:**
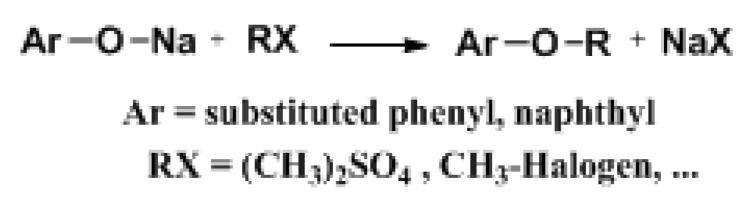
Conventional Williamson ether synthesis of alkyl aryl ethers.

The traditional Williamson ether synthesis, outlined in Figure 1, involves the S_N_2 reaction of an aryloxide with a primary alkyl halide, or of an E_AR_S reaction of an alkoxide with an aryl halide [7], in the presence [8,9] or absence of a solvent [10].

Though effective and generally considered to be the method of choice, the Williamson ether synthesis suffers from some drawbacks such as the toxicity of the associated alkyl halides and dimethyl and diethyl sulfate. In addition, some alkyl halides involved in this reaction are prone to elimination under basic reaction conditions. The Cu-promoted Ullmann ether synthesis [11] and its variants [8,12,13] are a useful alternative method, but is limited to certain reactive substrates and only takes place in high boiling polar solvents. The Mitsunobu protocol, whereby the relevant phenol and alcohol are condensed in the presence of diethyl azodicarboxylate and triphenylphosphine, smoothly allows access to the expected alkyl aryl ethers in good to excellent yields [14]. However the resultant triphenylphosphine oxide poses tremendous challenges during product purification. Recently, an alternative version of the Williamson ether synthesis using dialkyl carbonates and imidazole carbamates as the alkylating agents [4,15,16,17] has been proposed. Because BTE is mostly used as catalyst at low temperatures, its capacity to act as an alkylating agent has eluded the attention of organic chemists until now. We wish to report here our findings on its high temperature reaction with variously substituted phenols.

## 2. Results and Discussion

**Figure 2:**
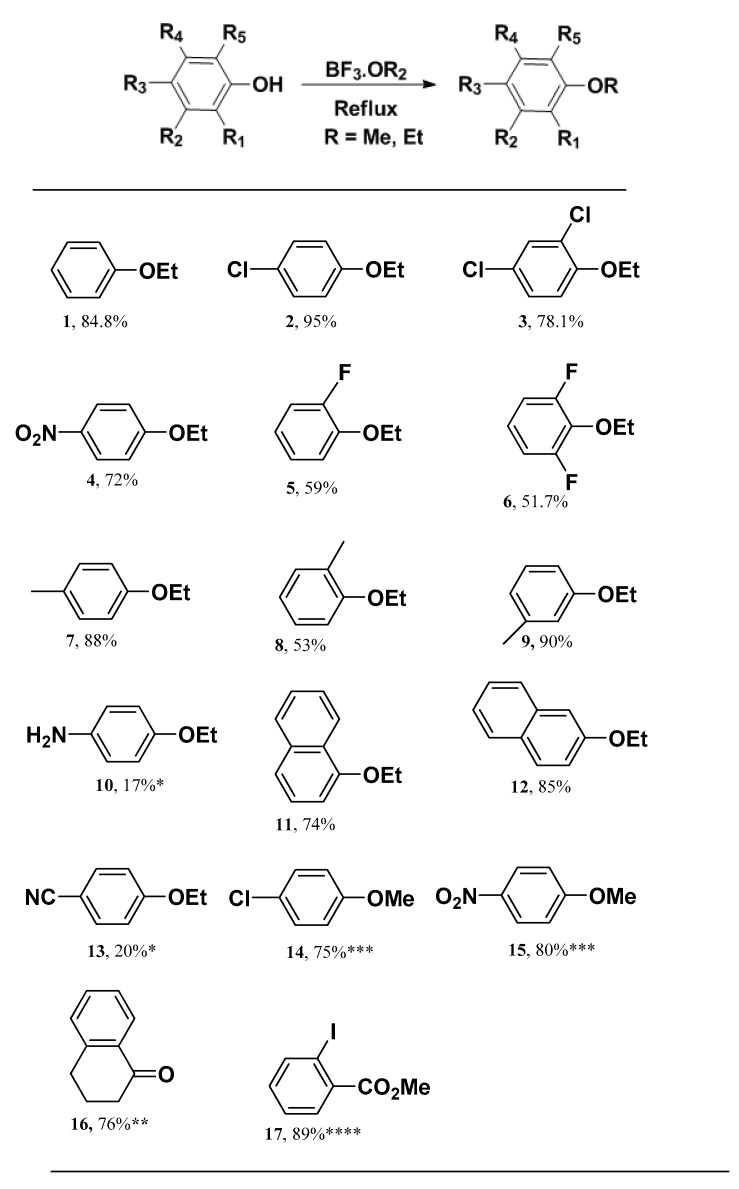
Alkylation of aryl alcohols by boron trifluoride etherates. * Reflux overnight. ** Reflux of 4-phenylbutyric acid with boron trifluoride methyl etherate. *** Reflux of the aryl alcohol in boron trifluoride methyl etherate. **** Reflux of o-iodobenzoic acid with boron trifluoride methyl etherate.

The results obtained in the reaction of various aryl alcohols with boron trifluoride etherate (BTE) are summarized in Figure 2 below.

Various aryl alcohols were ethylated under the above reaction conditions affording the corresponding ethers in low to excellent yield depending on the substitution on the aromatic ring. The reaction tolerated certain substituents such as, alkyl (compounds **7**–**9**), halogens (compounds **2**, **3**, **5**, and **6**), nitro (compound **4**), nitrile (compound **13**) and amino (compound **10**). The influence of some substituents, such amino, hydroxyl and nitro, on the overall outcome of the reaction depended on its nature and its position relative to the reaction center. Thus, while *p*-nitrophenol was converted to *p*-ethoxynitrobenzene (compound **4**), its ortho isomer (*o*-nitrophenol) failed to react under the same reaction conditions, most likely due to the steric hindrance brought about by the proximity of the nitro to the hydroxyl group. The amino group on the other hand showed a somewhat different trend: *p*-aminophenol was ethylated, albeit in low yield (compound **10**) after refluxing overnight. Its ortho isomer (*o*-aminophenol), on the other hand, formed the corresponding ether only in trace amounts and the slow reaction could also be attributed to the steric hindrance between the two adjacent groups (amino and hydroxyl). All three isomeric hydroxyphenols led to decomposition products, forming tarry materials. A nitrogen atom forming part of the aromatic ring system completely inhibited the ethylation reaction as exemplified by *p*-hydroxypyridine and 8-hydroxyquinoline, probably due to the chelation of the nitrogen atom onto the Lewis acid, leading to the formation of resinous materials. The presence of a nitrile group slowed down the ethylation reaction considerably (compound **13**) and only 20% of the anticipated product was obtained after refluxing the reaction overnight. As in all the cases where low yields are obtained, the starting material was not recovered and was completely converted into tars. All three isomers of hydroxybenzaldehyde decomposed and formed resinous materials. The decomposition of *p*-hydroxybenzyl alcohol, on the other hand, may not be surprising given that hydroxybenzyl alcohols are prone to polymerization under acidic conditions [18].

**Scheme 1:**
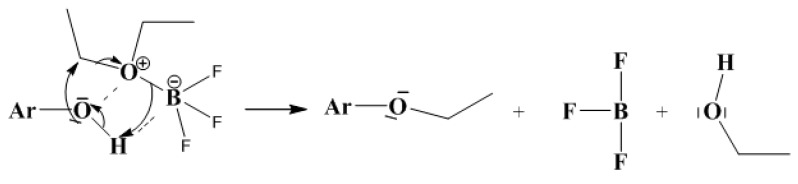
Proposed alkylation mechanism.

BTE promotes the formation of a carbocation with aliphatic hydroxyl groups, which subsequently undergo alkene formation if β-hydrogen atoms are present [19]. The alkenes formed in the process are likely to polymerize under the above reaction condition as observed in our previous investigations wherein undecylenic acid was esterified to ethyl undecylenate followed by the polymerization of the double bond [1]. Although the alkylation of carboxylic acids and ethylation of phenols were carried out in BTE as solvent, preliminary experiments done using this reagent in stoichiometric amounts without a solvent have proved to be equally successful. In the presence of solvents such as toluene and 1,2-dichloroethane the reactions were too slow to be of any significant value. The mechanism of the ethylation reaction is believed to involve the coordination of the oxygen atom of the hydroxyl group with BTE, followed by the transfer of the ethyl group by alkylation as depicted in Scheme 1.

A comparison between reported results of the synthesis of selected aryl ethyl ethers with the results obtained in the present work is shown in Table 1. The merit of this method lies in its novelty and simplicity, consisting merely in refluxing the substrate in BTE. 

Similar reactions using boron trifluoride methyl etherate with either carboxylic acids [1] and phenols produced similar results (see compounds **14**, **15**, and **17**), with the only notable exception of the reaction with 4-phenylbutyric acid, which led to the formation of α-tetralone, the product of the intramolecular Friedel-Crafts acylation in 76% yield. These results show that boron trifluoride methyl etherate is a stronger Lewis acid than BTE.

## 3. Materials and Methods

### General Information

Solvents (n-hexane, ethyl acetate) were purchased from Shalom Laboratory Supplies cc; TLC plates (silica 60) were obtained from DLD Scientific SA cc; boron trifluoride etherate and boron trifluoride dimethyl etherate were procured from Merck SA (Pty) Ltd; anhydrous sodium sulfate was obtained from Prestige Laboratory Supplies and anhydrous calcium chloride was purchased from United Scientific SA cc. All the above chemicals were used without further purification. Products were characterized by ^1^H- and ^13^C-NMR spectroscopy using a Varian spectrometer at 400 and 100 MHz for proton and carbon-13, respectively. Spectra were recorded from CDCl_3_ solutions employing TMS as an internal reference. All spectra are reported as δ (ppm) values. Please find the NMR spectra of compounds in Supplementary Materials.

General procedure (compound **1** [4,11]): In a 50 mL round-bottomed flask equipped with a magnetic stirring bar, a reflux condenser and a calcium chloride drying tube were placed phenol (1 g, 10.6 mmol) and boron trifluoride etherate (10 mL, 77 mmol). The reaction mixture was stirred and heated to 120 °C for four hours during which the progress of the reaction was monitored by thin layer chromatography (n-hexane/ethyl acetate 7:1). The cooled reaction mixture was carefully diluted with water (20 mL) and extracted with n-hexane. The aqueous phase was further extracted with n-hexane (20 mL). The combined organic extract was washed with water (10 mL) followed by a saturated solution of NaHCO_3_ (10 mL) until complete cessation of effervescence. The organic layer was separated and dried over anhydrous Na_2_SO_4_ and concentrated in vacuo affording a clear liquid, 1.23 g (94.9%). Column chromatography on silica gel (n-hexane/ethyl acetate 7:1) gave 1.1 g (84.8%) of a pleasant-smelling oil. δ_H_ (400 MHz, CDCl_3_): ^1^H NMR (400 MHz, Chloroform-d) δ 7.23–7.09 (m, 2H), 6.89–6.76 (m, 3H), 3.93 (q, J = 7.0 Hz, 2H), 1.32 (t, J = 7.0, 3H); ^13^C NMR (101 MHz, Chloroform-d) δ 158.93, 129.43, 120.53, 114.47, 63.27, 14.86.

Procedure for the preparation of α-tetralone (compound **16** [24]): A 50 mL round-bottomed flask equipped with a magnetic stirring bar, a reflux condenser and a calcium chloride drying tube was charged 4-phenylbutyric acid (1g, 6.1 mmol) and boron trifluoride dimethyl etherate (10 mL, 113 mmol). The reaction mixture was stirred and heated reflux for four hours during after which the thin layer chromatography analysis (n-hexane/ethyl acetate 5:1) showed that the reaction was complete. The cooled reaction mixture was carefully diluted with water (20 mL) and extracted with n-hexane. The aqueous phase was further extracted with n-hexane (20 mL). The combined organic extract was washed with water (10 mL) followed by a saturated solution of NaHCO_3_ (10 mL) until complete cessation of effervescence. The organic layer was separated and dried over anhydrous Na_2_SO_4_ and concentrated in vacuo affording a clear liquid which was purified by column chromatography on silica gel (n-hexane/ethyl acetate 5:1) to give clear liquid (0.622 g, 76%). ^1^H NMR (400 MHz, Chloroform-*d*) δ 8.00 (d, *J* = 7.7 Hz, 1H), 7.47–7.39 (t, *J* = 7.7 Hz, 1H), 7.27 (t, *J* = 7.7 Hz, 1H), 7.22 (d, *^J^* = 7.7 Hz, 1H), 2.93 (t, *J* = 6.0 Hz, 2H), 2.62 (t, *J* = 6.0 Hz, 2H), 2.10 (p, *J* = 6.4 Hz, 2H); ^13^C NMR (101 MHz, Chloroform-*d*) δ 198.22, 144.36, 133.26, 132.47, 128.65, 126.99, 126.47, 39.02, 29.56, 23.15.

*p-Ethoxychlorobenzene***2** [20]: clear liquid, 1.13 g (93%); ^1^H NMR (400 MHz, Chloroform-*d*) δ 7.17–7.08 (m, 2H), 6.75–6.67 (m, 2H), 3.88 (q, J = 7.0 Hz, 2H), 1.30 (t, *J* = 7.0 Hz, 3H); ^13^C NMR (101 MHz, Chloroform-*d*) δ 157.55, 129.24, 125.31, 115.72, 63.71, 14.72. 

*Ethoxy-2,4-dichlorobenzene***3** [25]: clear liquid, 0.91 g (78%); ^1^H NMR (400 MHz, Chloroform-*d*) δ 7.27 (d, *J* = 2.6 Hz, 1H), 7.07 (dd, *J* = 8.8, 2.6 Hz, 1H), 6.73 (d, *J* = 8.8 Hz, 1H), 3.98 (q, *J* = 7.0 Hz, 2H), 1.37 (t, *J* = 7.0 Hz, 3H); ^13^C NMR (101 MHz, Chloroform-d) δ 153.28, 129.89, 127.46, 125.42, 123.59, 113.96, 65.00, 14.59.

*p-Ethoxynitrobenzene***4** [10]: pale yellow solid, 0.87 g (72%); ^1^H NMR (400 MHz, Chloroform-*d*) δ 8.18 (d, *J* = 9.1 Hz, 2H), 6.93 (d, *J* = 9.1 Hz, 2H), 4.12 (q, *J* = 6.9 Hz, 2H), 1.45 (t, *J* = 7.0 Hz, 3H). ^13^C NMR (101 MHz, Chloroform-*d*) δ 164.03, 125.92, 114.35, 64.40, 14.55.

*o-Ethoxyfluorobenzene***5** [26]: clear liquid, 0.74 g (59%); ^1^H NMR (400 MHz, Chloroform) δ 6.86–7.11 (m, 4H), 4.11 (q, *J* = 7.0 Hz, 2H), 1.46 (t, *J* = 7.0 Hz, 3H);^13^C NMR (101 MHz, Chloroform-*d*) δ 152.75 (d, *J_C-F_* = 245.25), 146.95 (d, J_C-F_ = 10.12), 124.20 (d, *J_C-F_* = 3.89), 120.85 (d, *J_C-F_* = 7.00), 116.08 (d, *J_C-F_* = 17.91), 114.84 (d, *J_C-F_* = 1.55), 64.80, 14.75.

*Ethoxy-2,6-difluorobenzene***6** [27]: clear liquid, 0.63 g (51.7%); ^1^H NMR (400 MHz, Chloroform-d) δ 6.98–6.73 (m, 3H), 4.18 (qm, *J* = 7.0, 2H), 1.37 (t, *J* = 7.0, 3H); ^13^C NMR (101 MHz, Chloroform-d) δ 156.41 (dd, *J_C-F_* = 248.34, 5.45), 135.44 (t, *J_C-F_* = 14.41), 122.60 (t, *J_C-F_* = 9.34), 112.04 (dd, *J_C-F_* = 16.35, 6.23), 64.80, 14.75.

*p-Ethoxytoluene***7** [21]: clear liquid, 1.11 g (88%); ^1^H NMR (400 MHz, Chloroform-*d*) δ 7.02–6.95 (m, 2H), 6.75–6.67 (m, 2H), 3.91 (q, J = 7.0, 2H), 2.19 (s, 3H), 1.31 (t, *J* = 7.0, 3H); ^13^C NMR (101 MHz, Chloroform-*d*) δ 156.76, 129.82, 129.64, 114.28, 63.36, 20.41, 14.85.

*o-Ethoxytoluene***8** [21]: clear liquid, 0.67 g (53%); ^1^H NMR (400 MHz, Chloroform-*d*) δ 7.56–7.46 (m, 2H), 7.26–7.13 (m, 2H), 4.39 (q, *J* = 7.0 Hz, 2H), 2.61 (s, 3H), 1.79 (t, *J* = 7.0 Hz, 3H); ^13^C NMR (101 MHz, Chloroform-*d*) δ 159.02, 139.40, 129.18, 121.38, 115.40, 111.38, 63.23, 21.52, 14.90.

*m-Ethoxytoluene***9** [4]: clear liquid, 1.13 g (90%); ^1^H NMR (400 MHz, Chloroform-*d*) δ 7.19 (t, *J* = 8.1 Hz, 1H), 6.56–6.46 (m, 3H), 4.03 (q, *J* = 7.0 Hz, 2H), 3.80 (s, 3H), 1.43 (t, *J* = 7.0 Hz, 3H); ^13^C NMR (101 MHz, Chloroform-*d*) δ 159.02, 139.40, 129.18, 121.38, 115.40, 111.38, 63.23, 21.52, 14.90.

*p-Ethoxyaniline***10** [28]: light yellow liquid, 0. 21 g (17%); ^1^H NMR (400 MHz, Chloroform) δ 6.69–6.62 (m, 2H), 6.55 (d, J = 8.7 Hz, 2H), 3.87 (q, J = 7.0 Hz, 2H), 3.26 (bs, 2H), 1.29 (t, *J* = 7.0 Hz, 3H); ^13^C NMR (101 MHz, Chloroform-*d*) δ 152.13, 139.79, 116.44, 115.67, 64.04, 14.95.

*α-Ethoxynaphthalene***11** [28]: clear liquid, 0.88 g (74%);^1^H NMR (400 MHz, Chloroform-*d*) δ 7.64–7.82 (m, 3H), 7.47–7.51 (m, 1H), 7.36–7.40 (m, 1H), 7.16–7.23 (m, 2H), 4.26 (q, *J* = 6.85 Hz, 2H), 1.64 (t, J = 6.85 Hz, 3H); ^13^C NMR (101 MHz, Chloroform-*d*) δ 156.9, 134.59, 129.29, 128.87, 127.59, 126.67, 126.23, 123.44, 118.96, 106.51, 63.35, 14.75.

*β-Ethoxynaphthalene***12** [4,23]: clear liquid, 1.01 g (85%);^1^H NMR (400 MHz, Chloroform-*d*) δ 8.47–8.50 (m, 1H), 7.91–7.93 (m, 1H), 7.46–7.63 (m, 4H), 6.87 (d, J = 7.83 Hz, 1H), 4.26 (q, *J* = 7.0 Hz, 2H), 1.64 (t, J = 7.0 Hz, 3H); ^13^C NMR (101 MHz, Chloroform-*d*) δ 154.70, 134.47, 127.35, 126.23, 125.85, 125.70, 124.96, 122.06, 119.93, 104.51, 63.55, 14.74.

*p-Ethoxybenzonitrile***13** [29]: white solid, 0.25 g (20%); ^1^H NMR (400 MHz, Chloroform) δ 7.54–7.46 (m, 2H), 6.90–6.82 (m, 2H), 4.01 (q, J = 7.0 Hz, 2H), 1.37 (t, *J* = 7.0 Hz, 3H); ^13^C NMR (101 MHz, Chloroform-*d*) δ 162.23, 133.94, 119.27, 115.13, 103.69, 63.90, 14.53.

*p-Chloroanisole***14** [30]: colourless liquid, 0.82 g (75%);^1^H NMR (400 MHz, Chloroform) δ 7.24 (d, *J* = 8.7 Hz, 2H), 6.83 (d, *J* = 8.7 Hz, 2H), 3.78 (s, 3H). ^13^C NMR (101 MHz, Chloroform-*d*) δ 158.10, 129.23, 125.43, 115.06, 55.40.

*p-Nitroanisole***15** [31]: white solid, 0.88 g (80%);^1^H NMR (400 MHz, Chloroform) δ 8.17 (d, *J* = 9.2 Hz, 2H), 6.93 (d, *J* = 9.2 Hz, 2H), 3.89 (s, 3H). ^13^C NMR (101 MHz, Chloroform-*d*) δ 164.52, 141.51, 125.79, 113.84, 55.78.

*Methyl o-iodobenzoate***17** [32]; colorless liquid, 0.9441g (89% yield. ^1^H NMR (400 MHz, Chloroform) δ 7.95 (d, *J* = 7.83 Hz, 1H), 7.76 (d, *J* = 7.44 Hz, 1H), 7.36 (t, *J* = 7.63 Hz, 1H), 3.90–7.11 (t, *J* = 7.44 Hz, 1H). ^13^C NMR (101 MHz, Chloroform-*d*) δ 166.80, 141.17, 134.88, 132.56, 130.82, 127.79, 94.00, 55.20. 

## 4. Conclusions

This reaction compares more favorably in terms of cost and simplicity to other potential processes, especially those listed in Table 1 above. 

While the high cost of BTE may be a hindrance to a wide scale industrial application of this ethylation protocol compared to the traditional Williamson ether synthesis, it may be of use in the pharmaceutical and fine chemical industries, where batch reactions are widely in operation and emphasis is on small production volumes. 

## Figures and Tables

**Table 1:** Comparison of the results obtained for the synthesis of aryl ethyl ethers with those recently reported in the literature.

Product	This Work (%)	Literature Conditions	Literature Yield (%)	Reference
Phenetole **1**	84.8	Phenol/EtONa/diethyl carbonate, 137 °C/42 h.	71	[4]
*p*-Chloroethoxybenzene **2**	95	*p*-Chlorophenol/K_2_CO_3_/EtBr/Acetone, reflux, 3 h.	89	[20]
*p*-Ethoxytoluene **11**	88	*p*-Iodotoluene/NMP, 170 °C/Cu_2_O (5%), CsCO_3_ (2 eqv), MW, 30 min.	77	[21]
*p*-Nitroethoxybenzene **4**	72	*p*-Fluoronitrobenzene/EtOH, Triton B/50 °C, 20 h.	93	[22]
β-Ethoxynaphthalene **18**	85	2-naphthol/EtBr/NaOH 40%/TBAB/Toluene, 70 °C, 4 h.	98.1	[23]

## Supplementary Materials

The NMR spectra of compounds obtained in this study are available online.
