# Supplementary material for: A New Alkylation of Aryl Alcohols by Boron Trifluoride Etherate
Source: Molecules. 2019 Oct 16;24(20):3720. doi: 10.3390/molecules24203720 (PMC6833367; doi:10.3390/molecules24203720)
Supplement: Supplementary file 1 [file molecules-24-03720-s001.pdf]

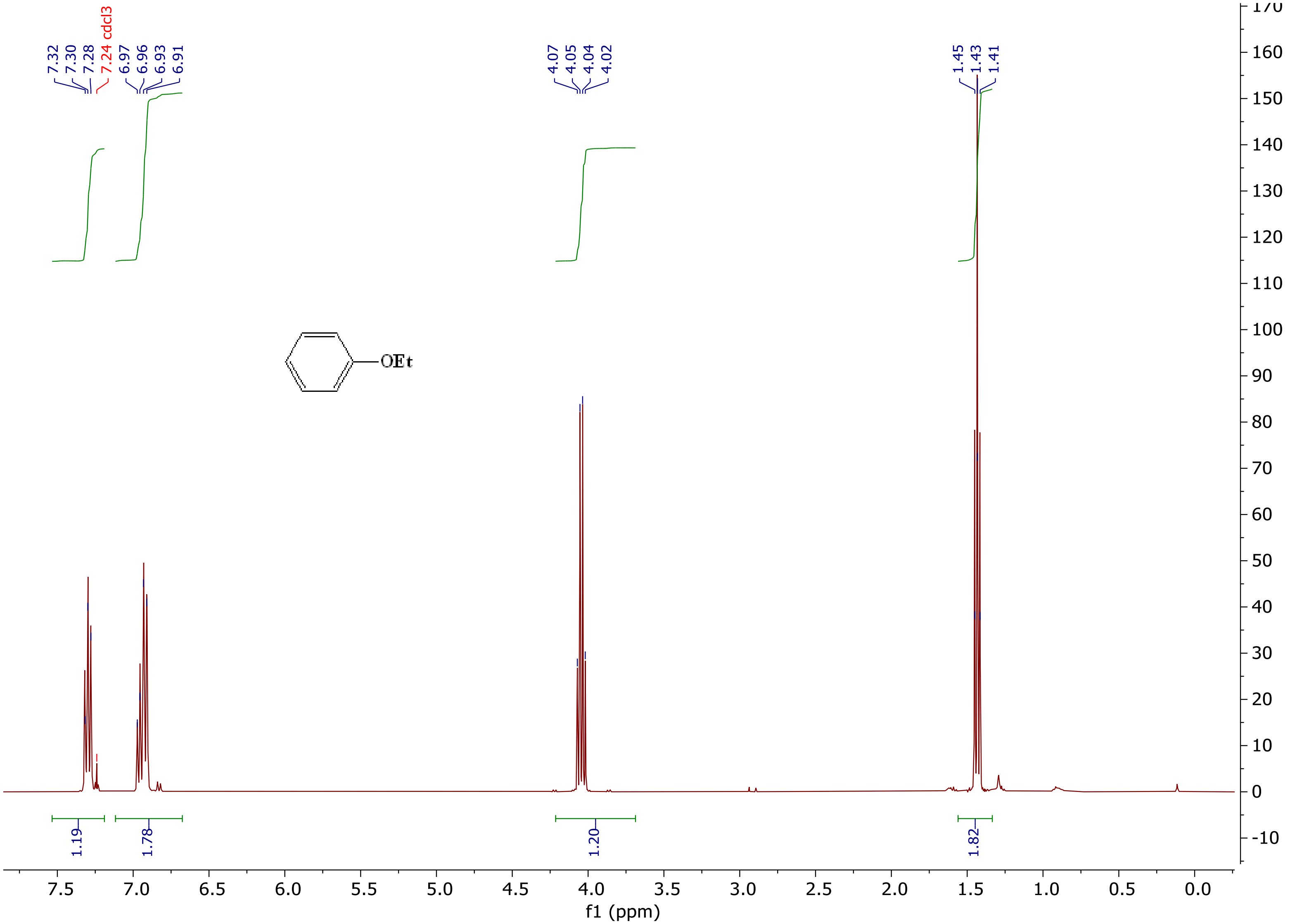

S1: ^{1}H-NMR spectrum of ethoxybenzene **1**.

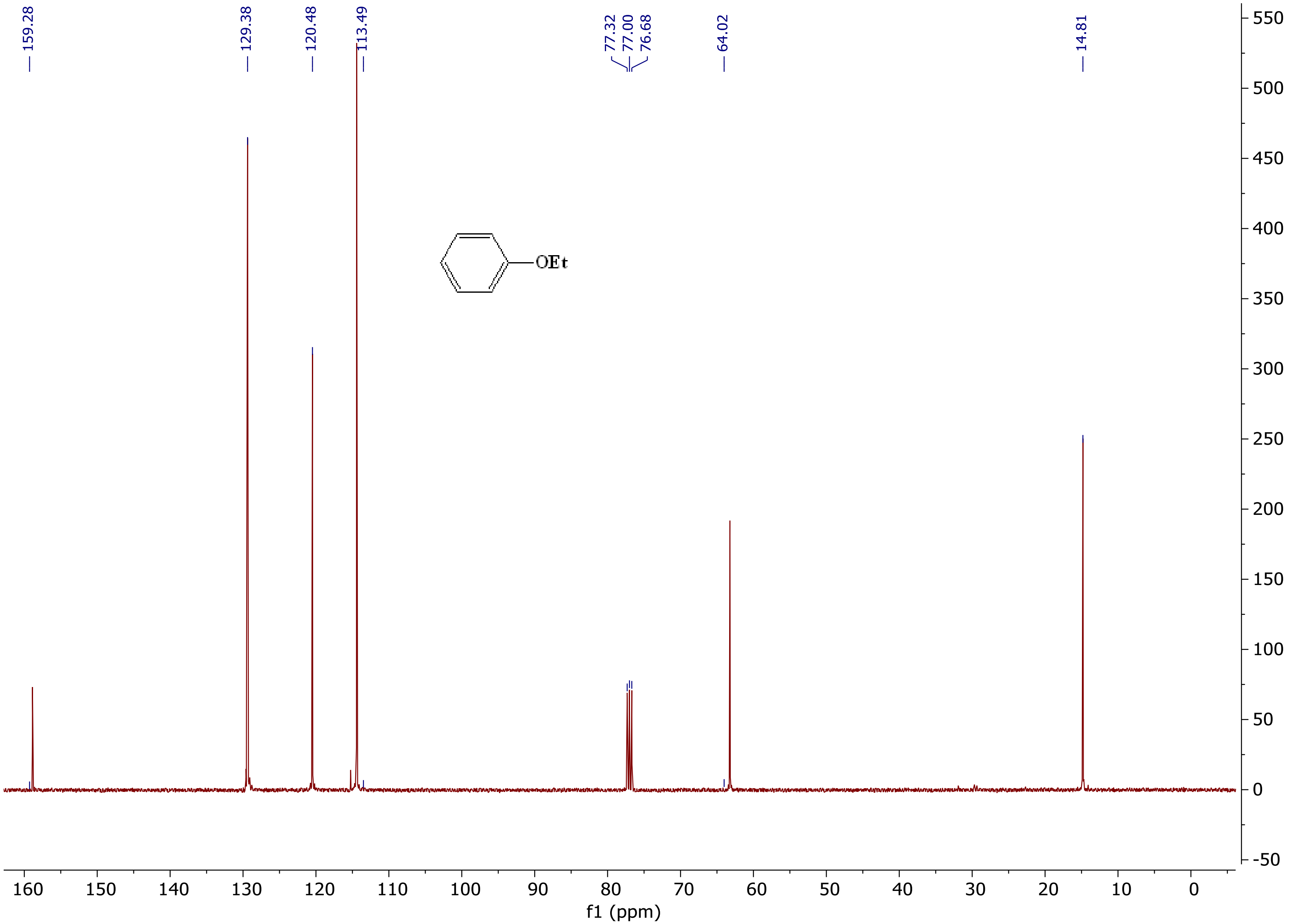

S1: ^{13}C-NMR spectrum of ethoxybenzene **1**.

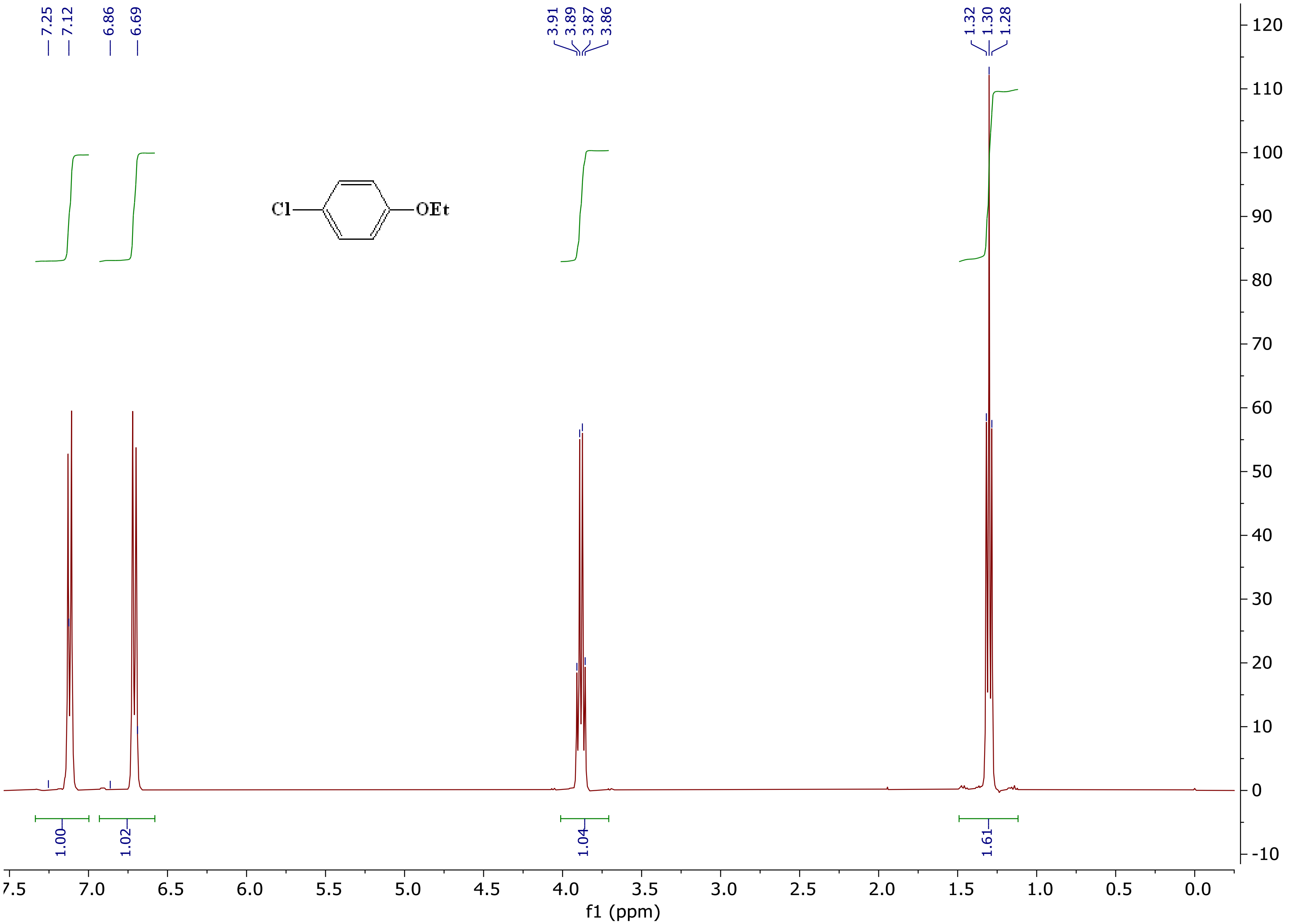

S3: ^{1}H-NMR spectrum of p-chloroethoxybenzene **2**.

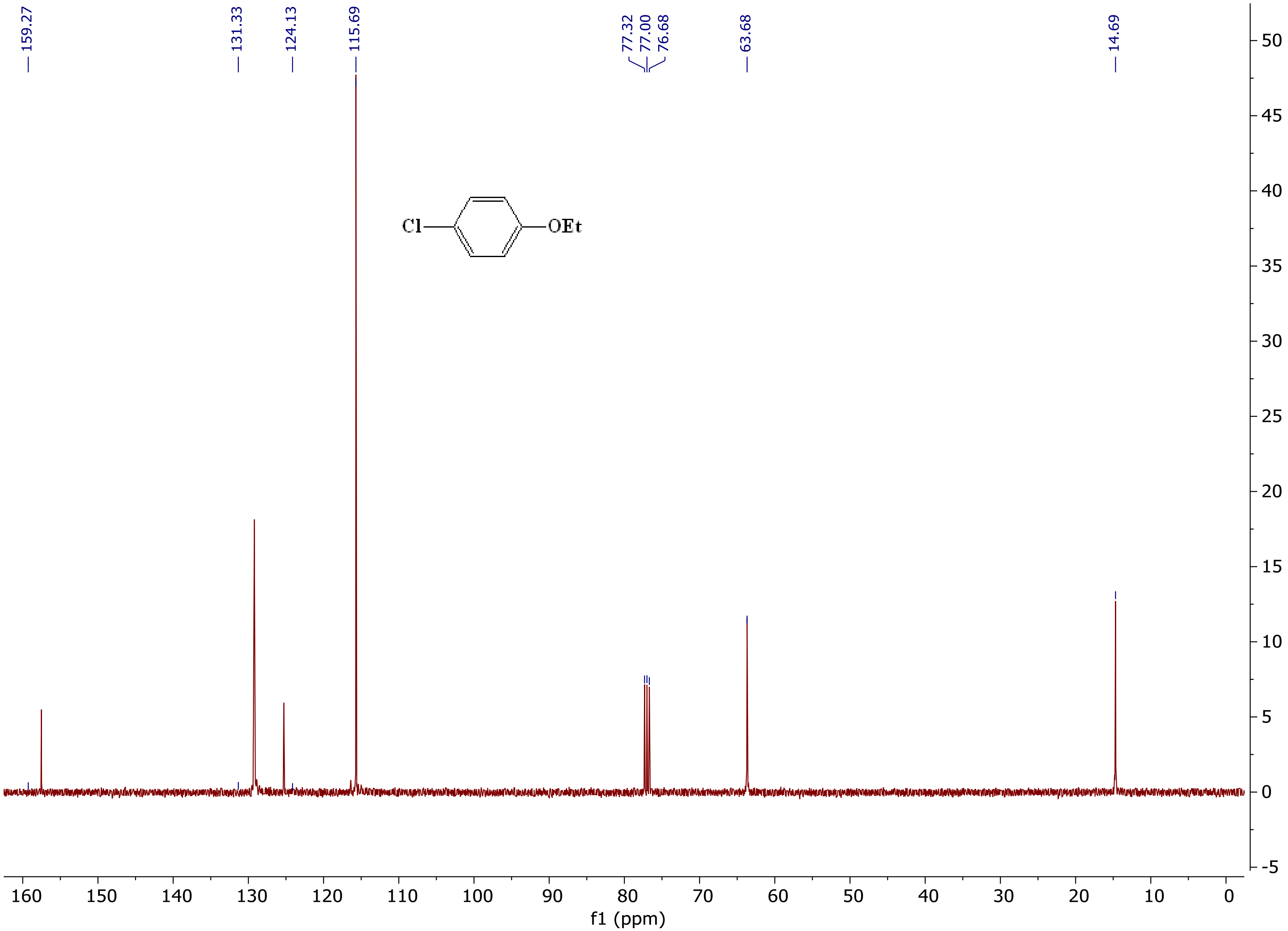

S4: ^{13}C-NMR spectrum of p-chloroethoxybenzene **2**.

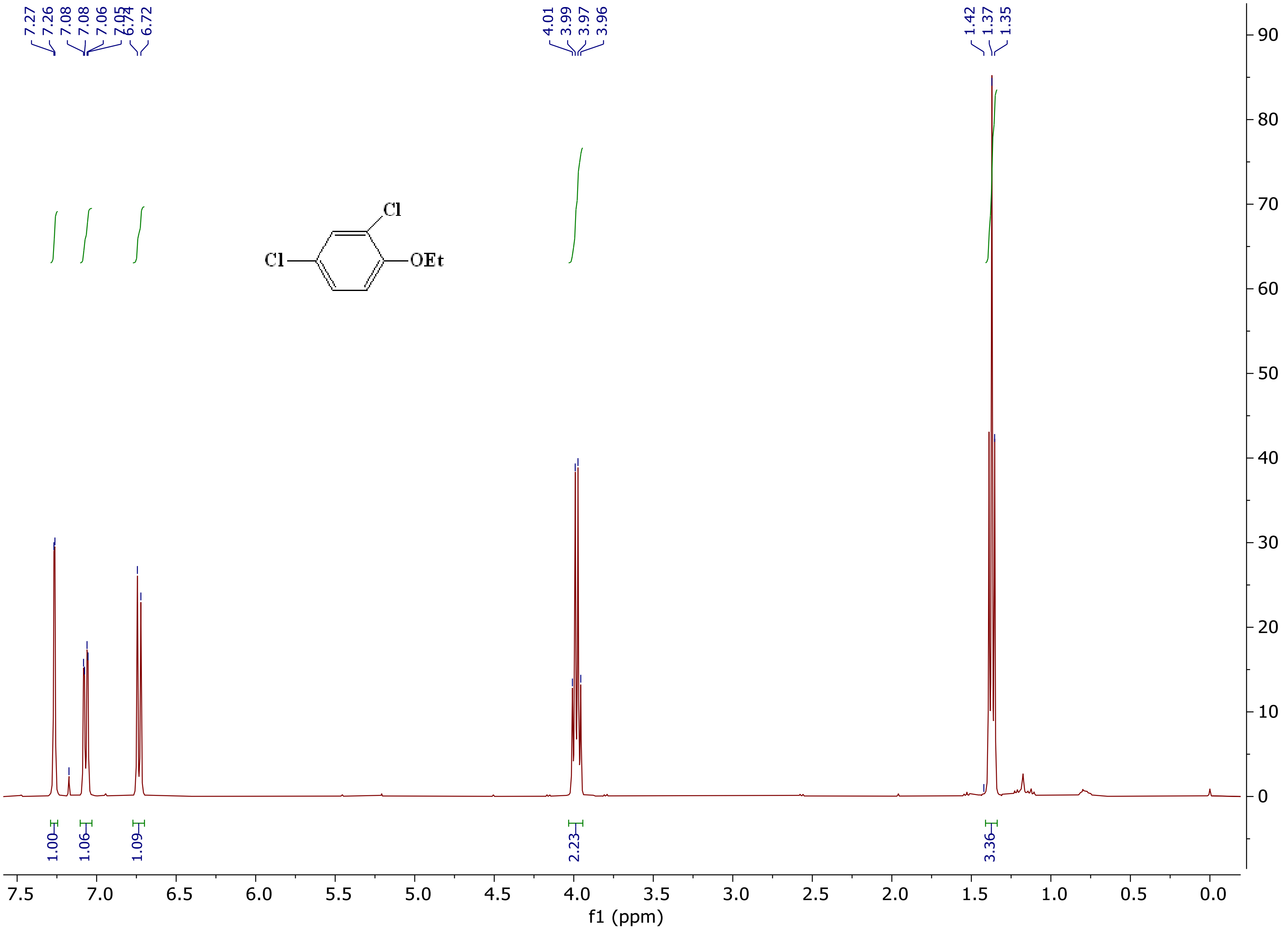

S5: ^{1}H-NMR spectrum of 2,4-dichloroethoxybenzene **3.**

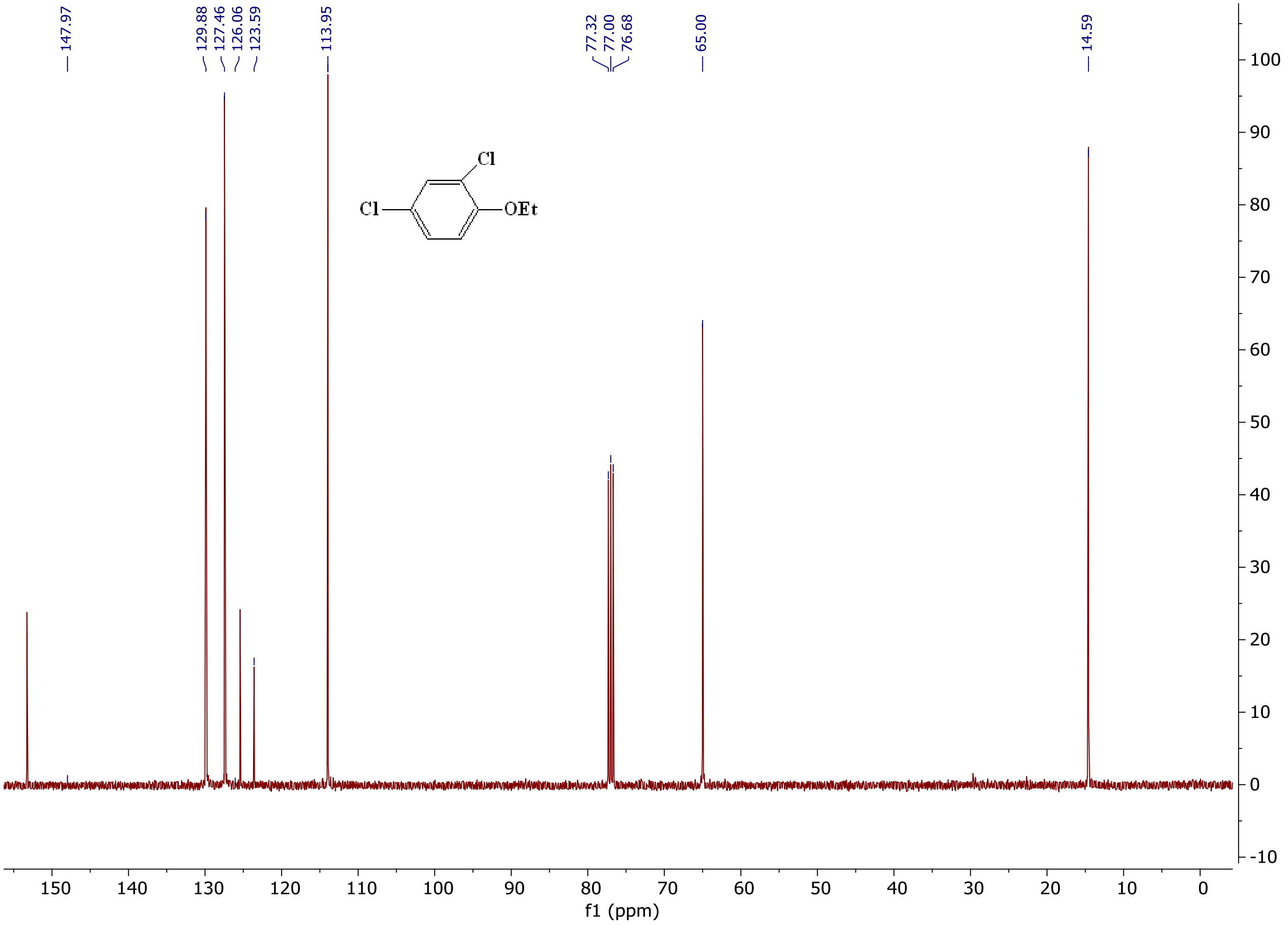

S6: ^{13}C-NMR spectrum of 2,4-dichloroethoxybenzene **3.**

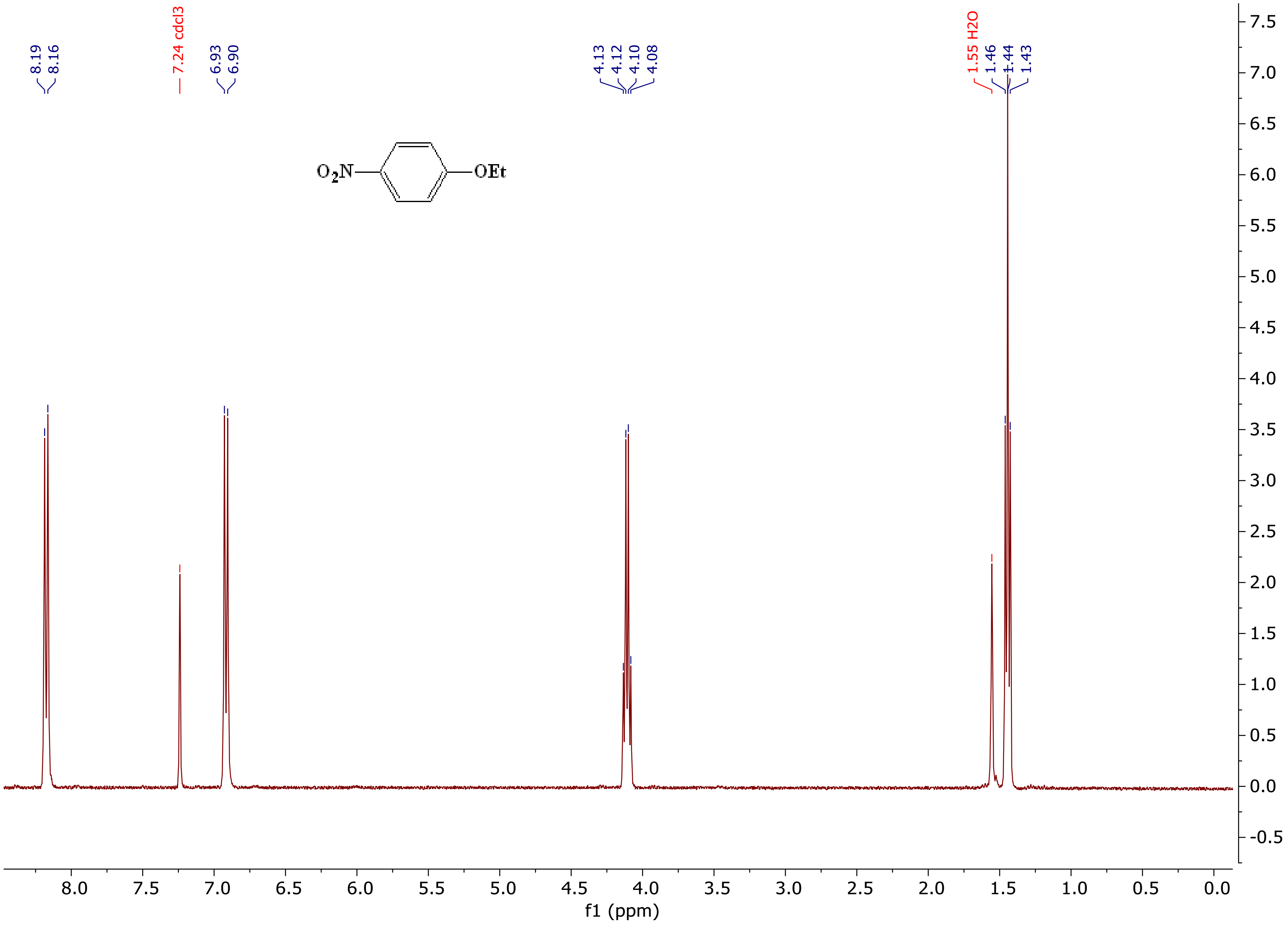

S7: ^{1}H-NMR spectrum of *p*-ethoxynitrobenzene **4.**

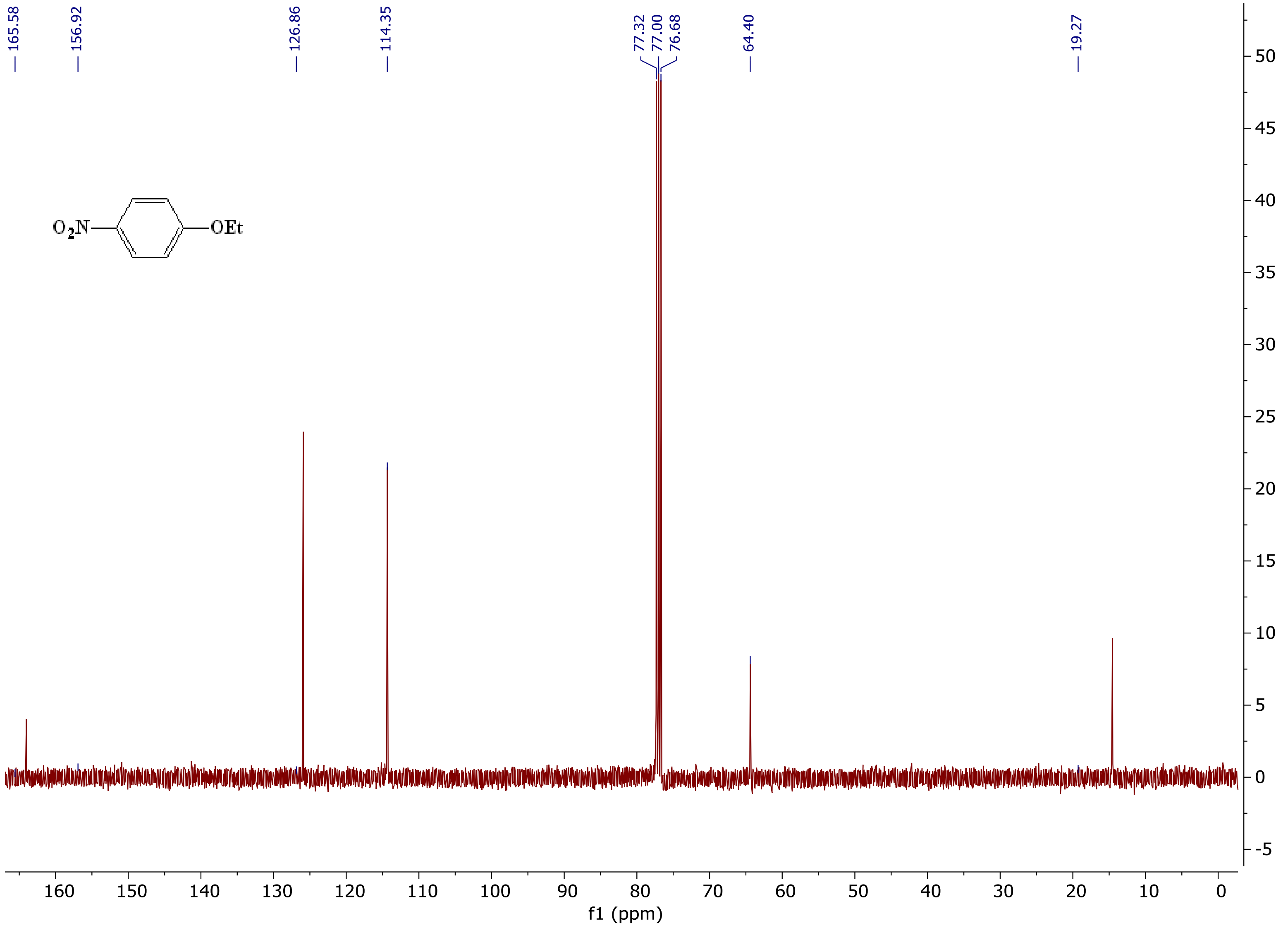

S8: ^{13}C-NMR spectrum of *p*-ethoxynitrobenzene **4.**

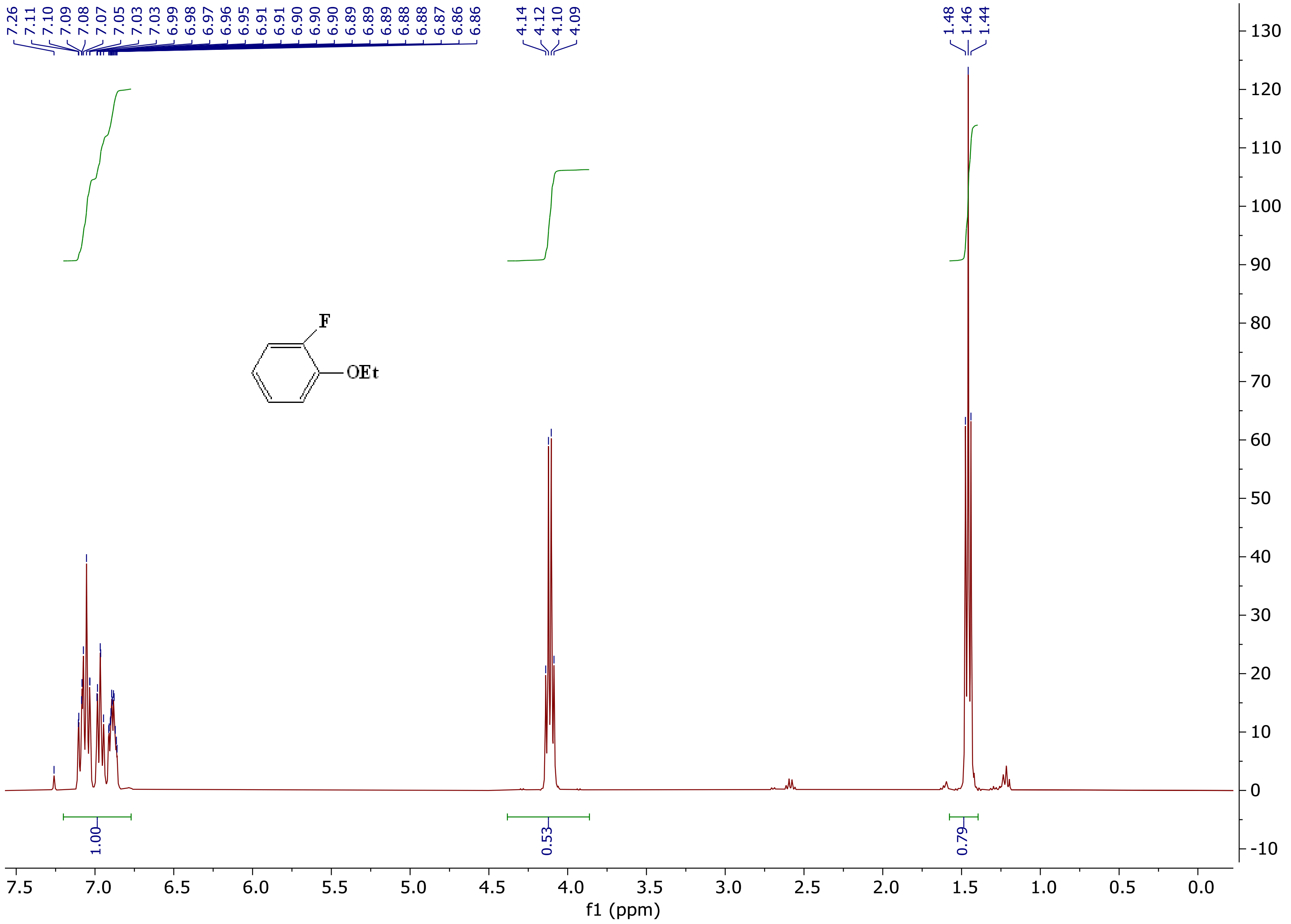

S9: ^{1}H-NMR spectrum of *o*-ethoxyfluorobenzene **5.**

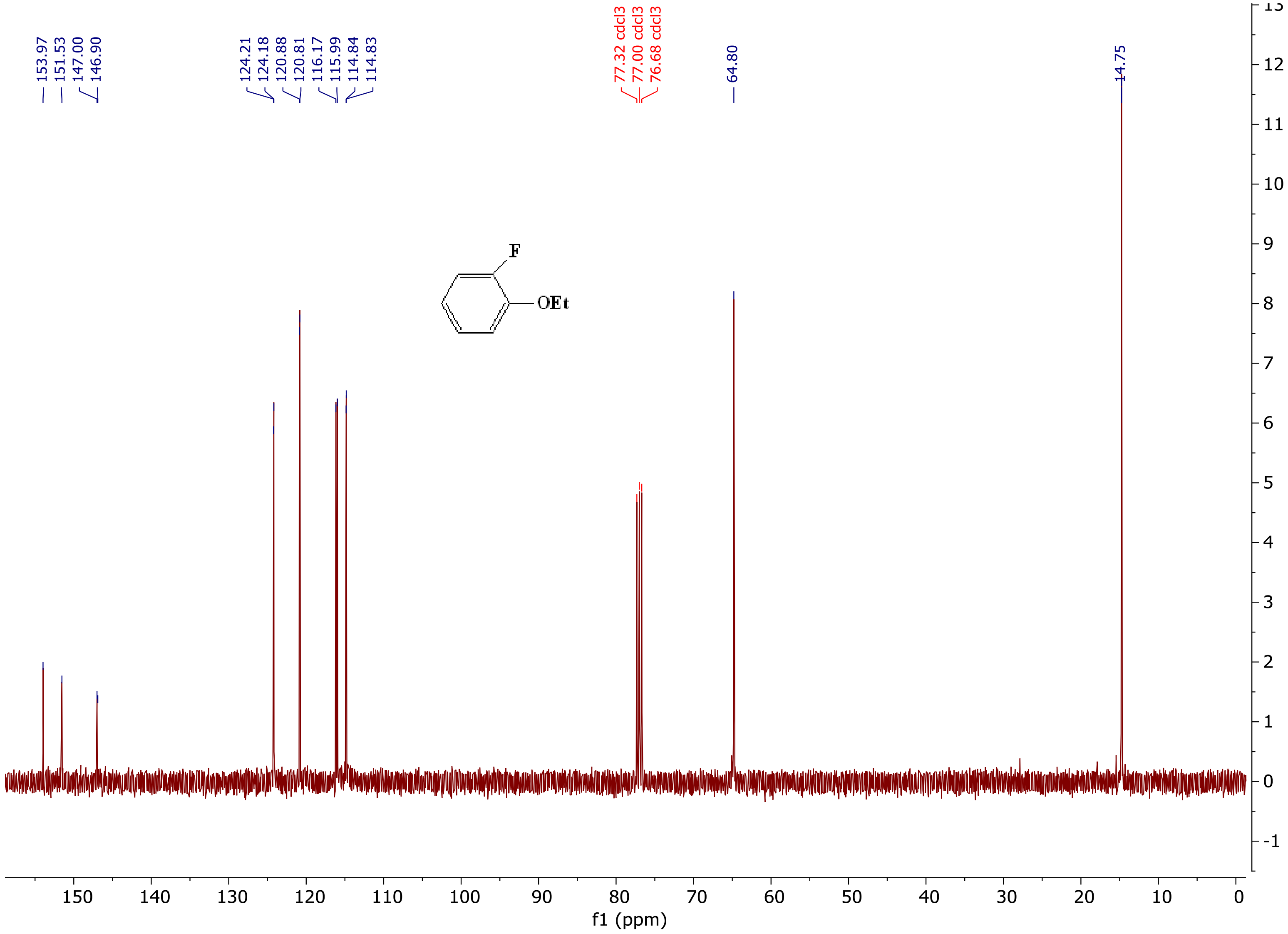

S10: ^{13}C-NMR spectrum of *o*-ethoxyfluorobenzene **5.**

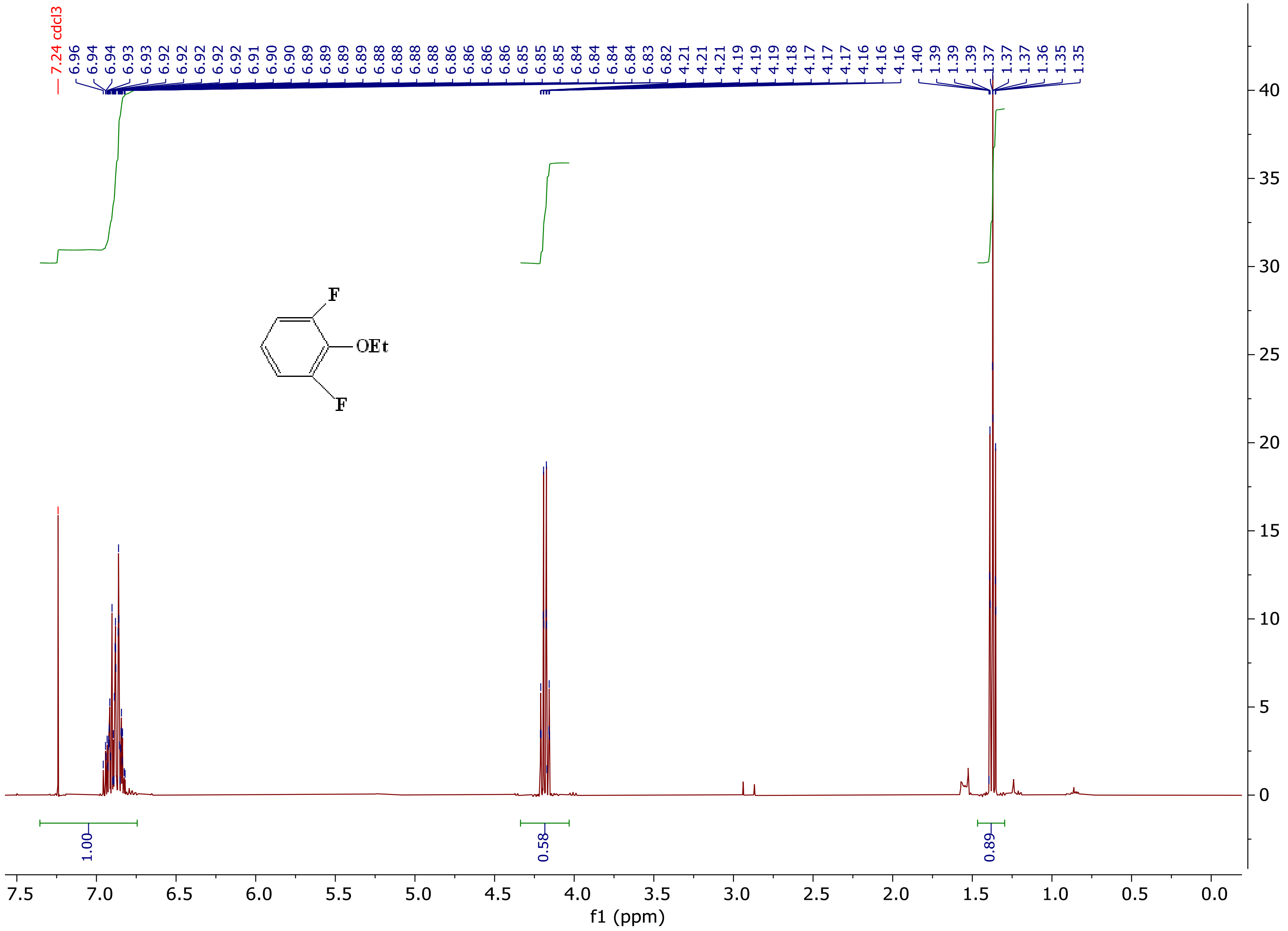

S11: ^{1}H-NMR spectrum of 2,6-difluoroethoxybenzene **6.**

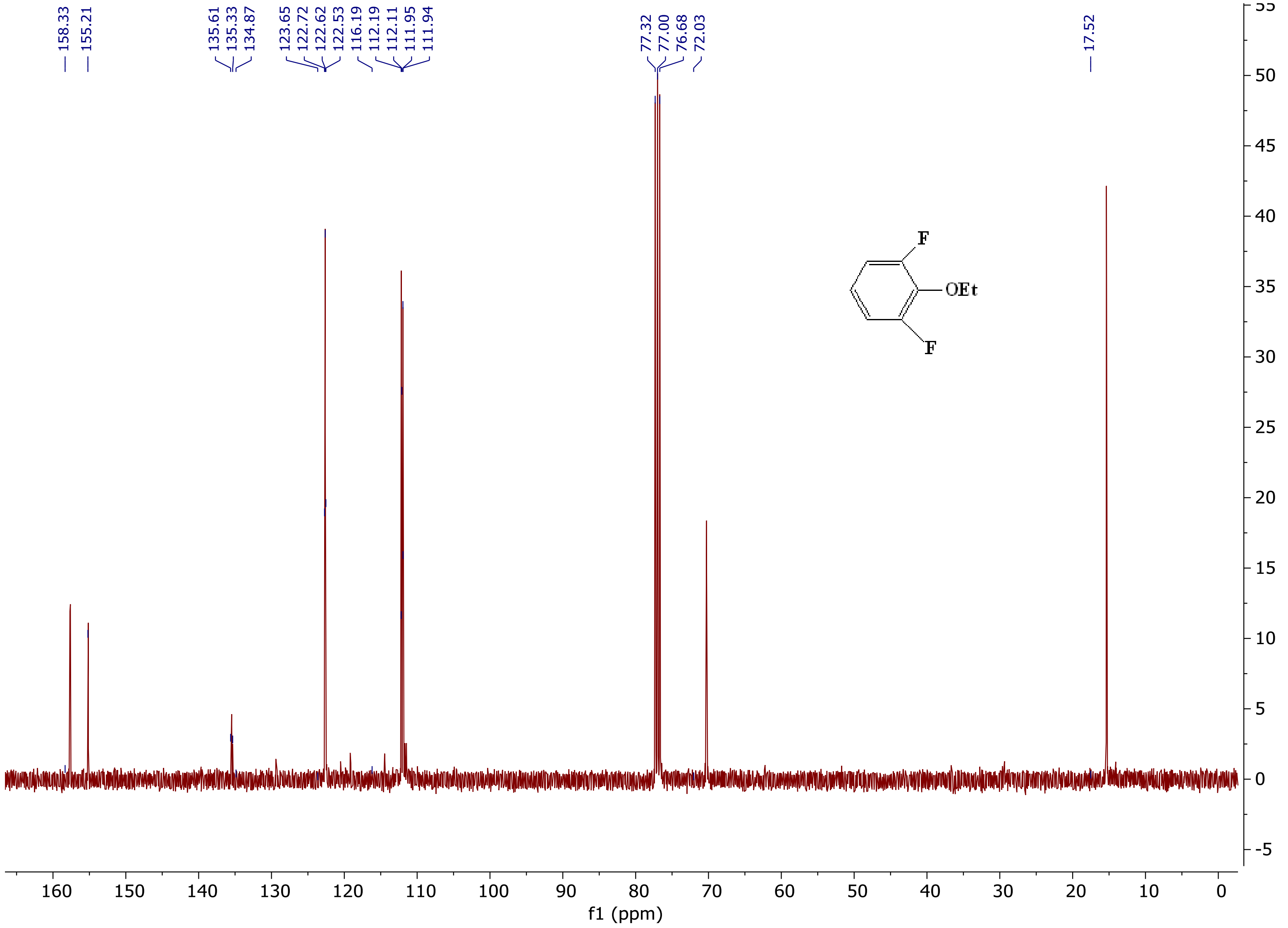

S12: ^{13}C-NMR spectrum of 2,6-difluoroethoxybenzene **6.**

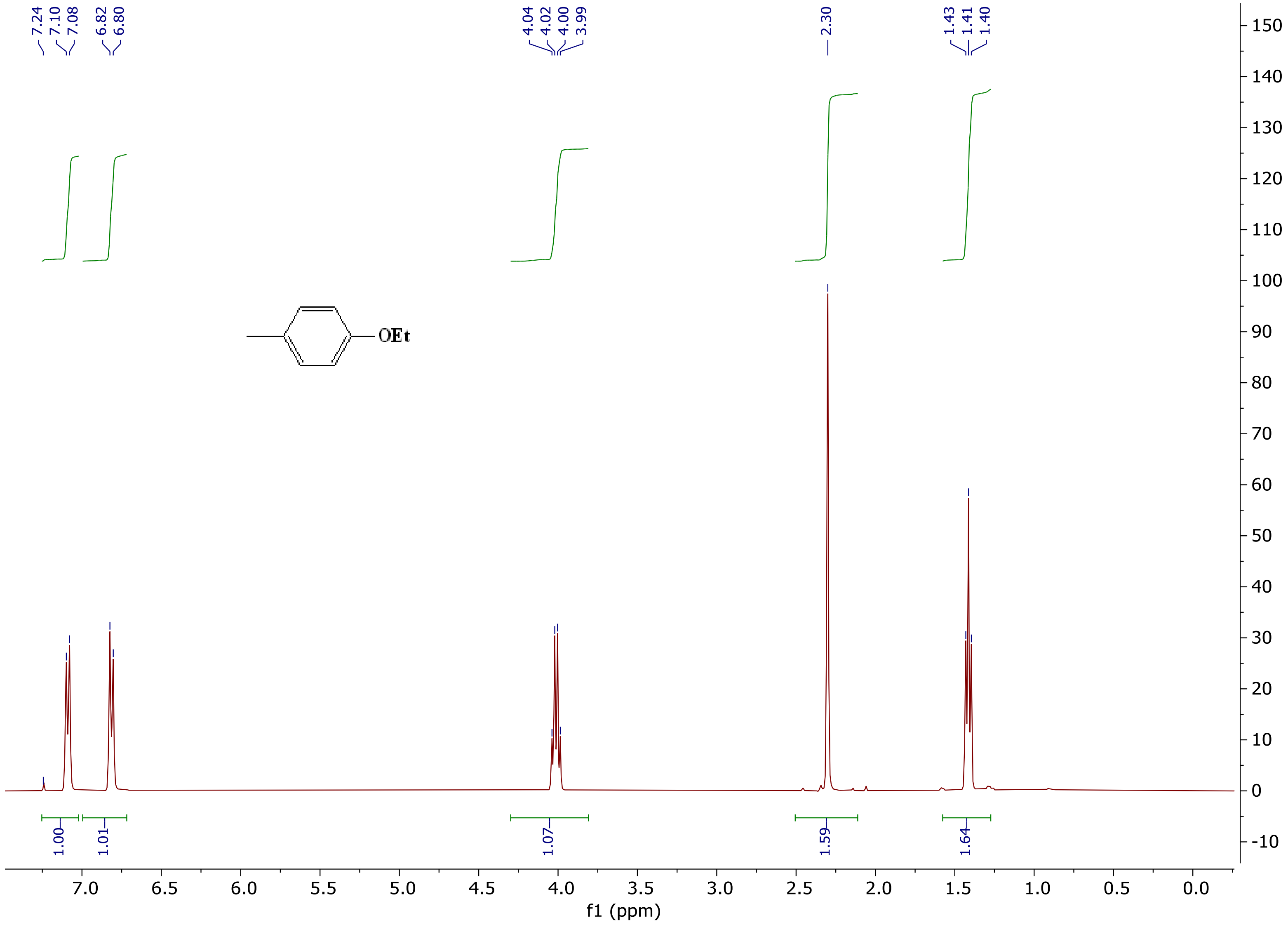

S13: ¹H-NMR spectrum of *p*-ethoxytoluene **7.**

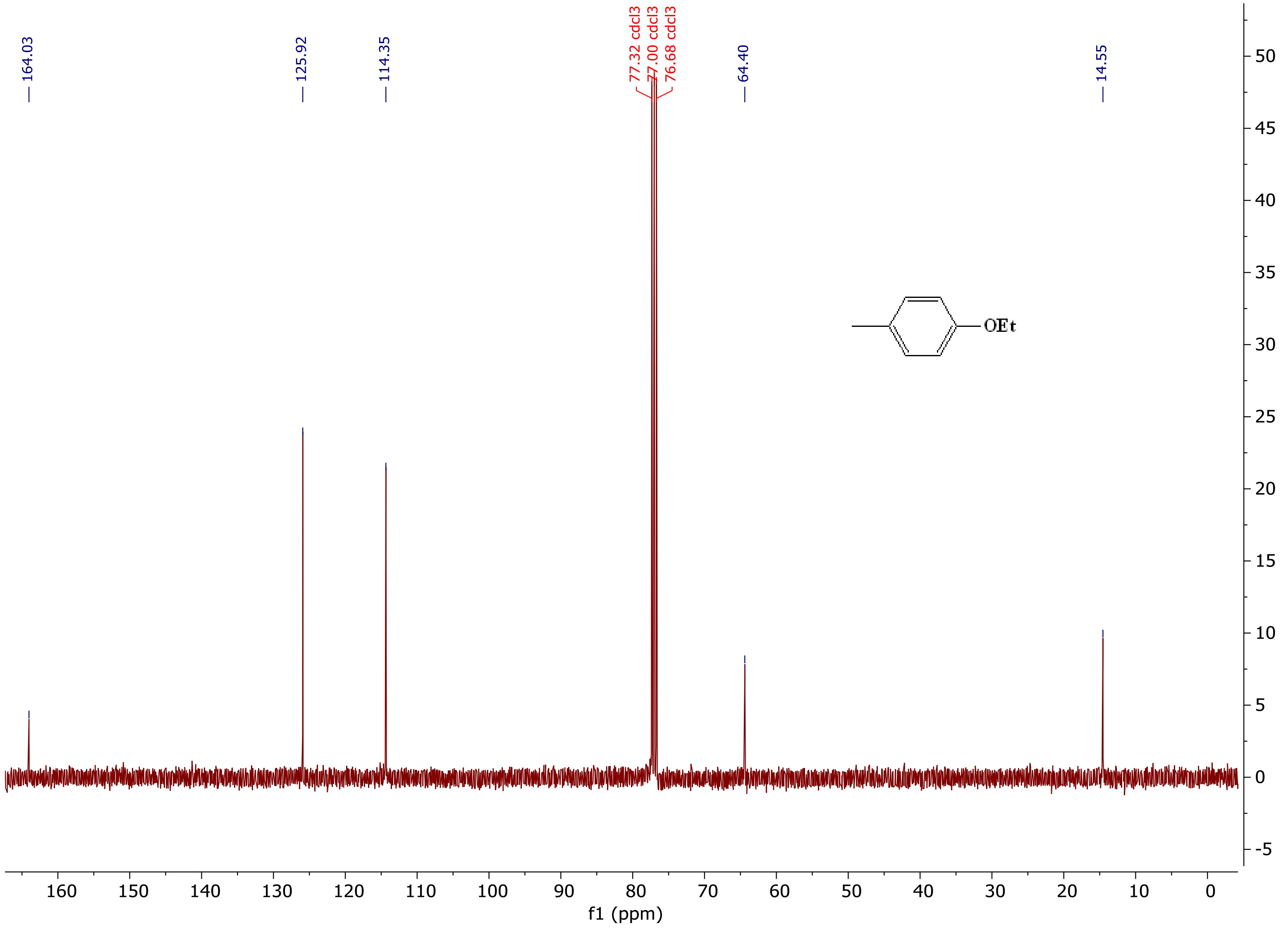

S14: ^{13}C-NMR spectrum of *p*-ethoxytoluene **7.**

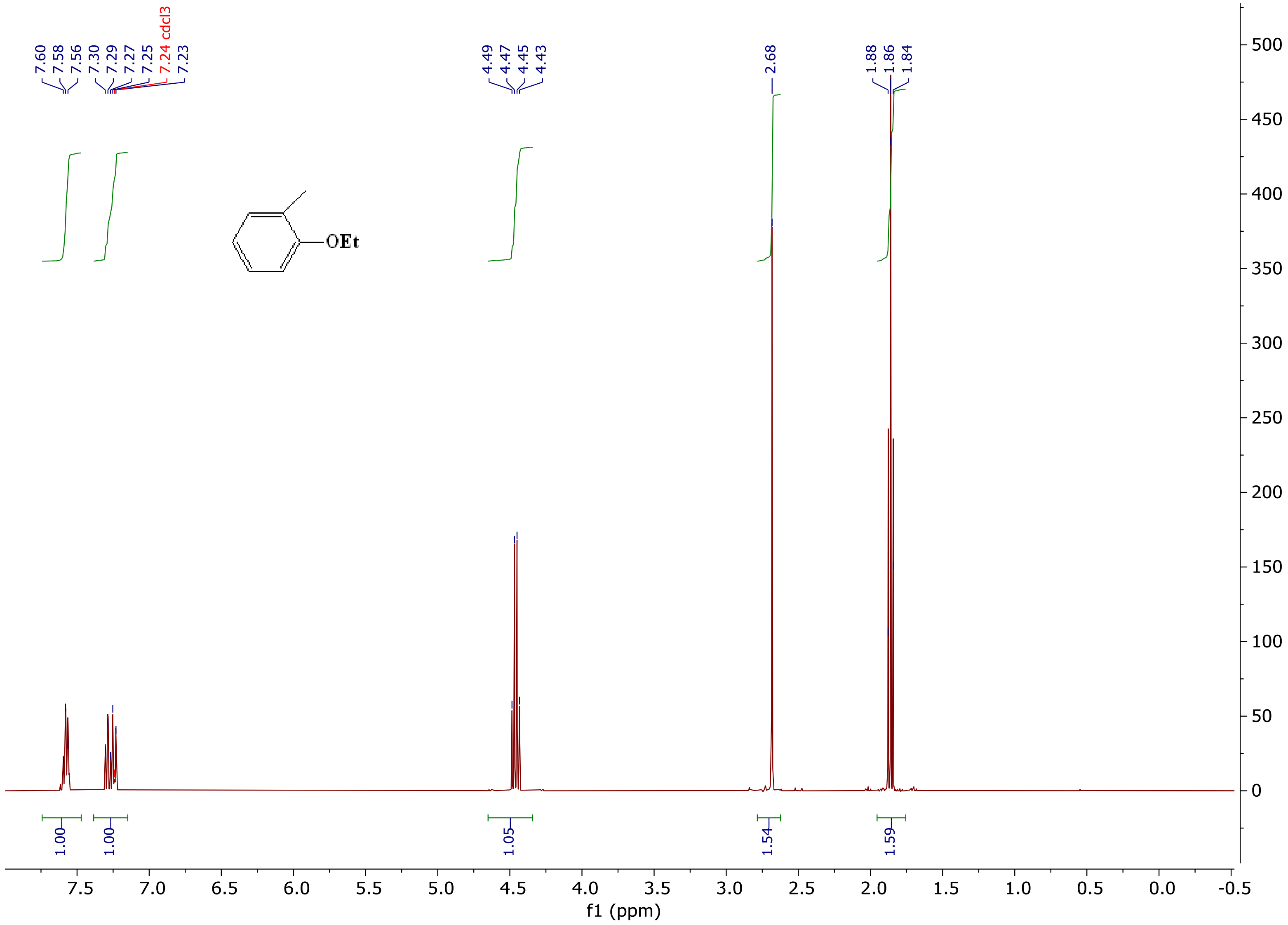

S15: ^{1}H-NMR spectrum of *o*-ethoxytoluene **8.**

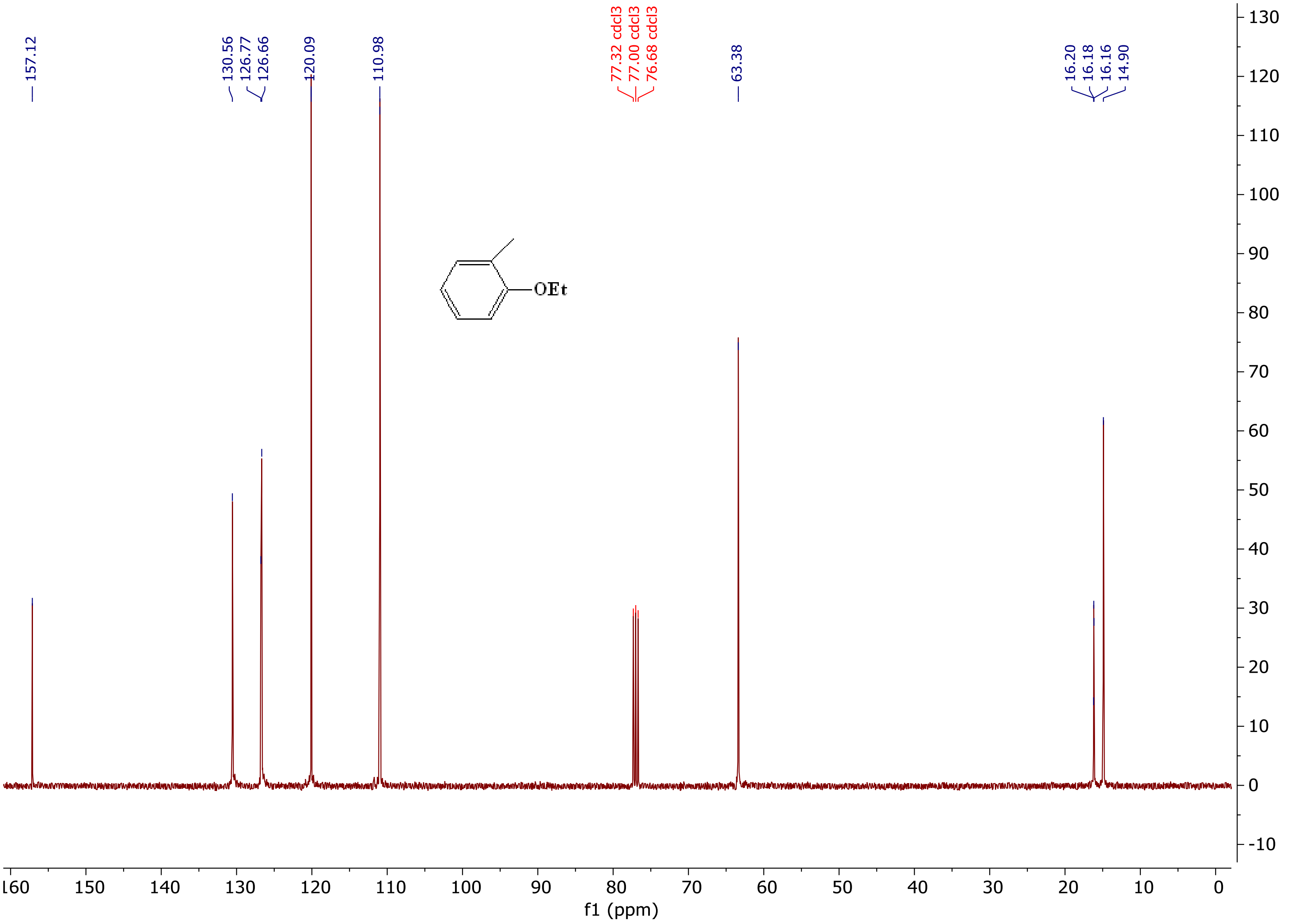

S16: ^{13}C-NMR spectrum of *o*-ethoxytoluene **8.**

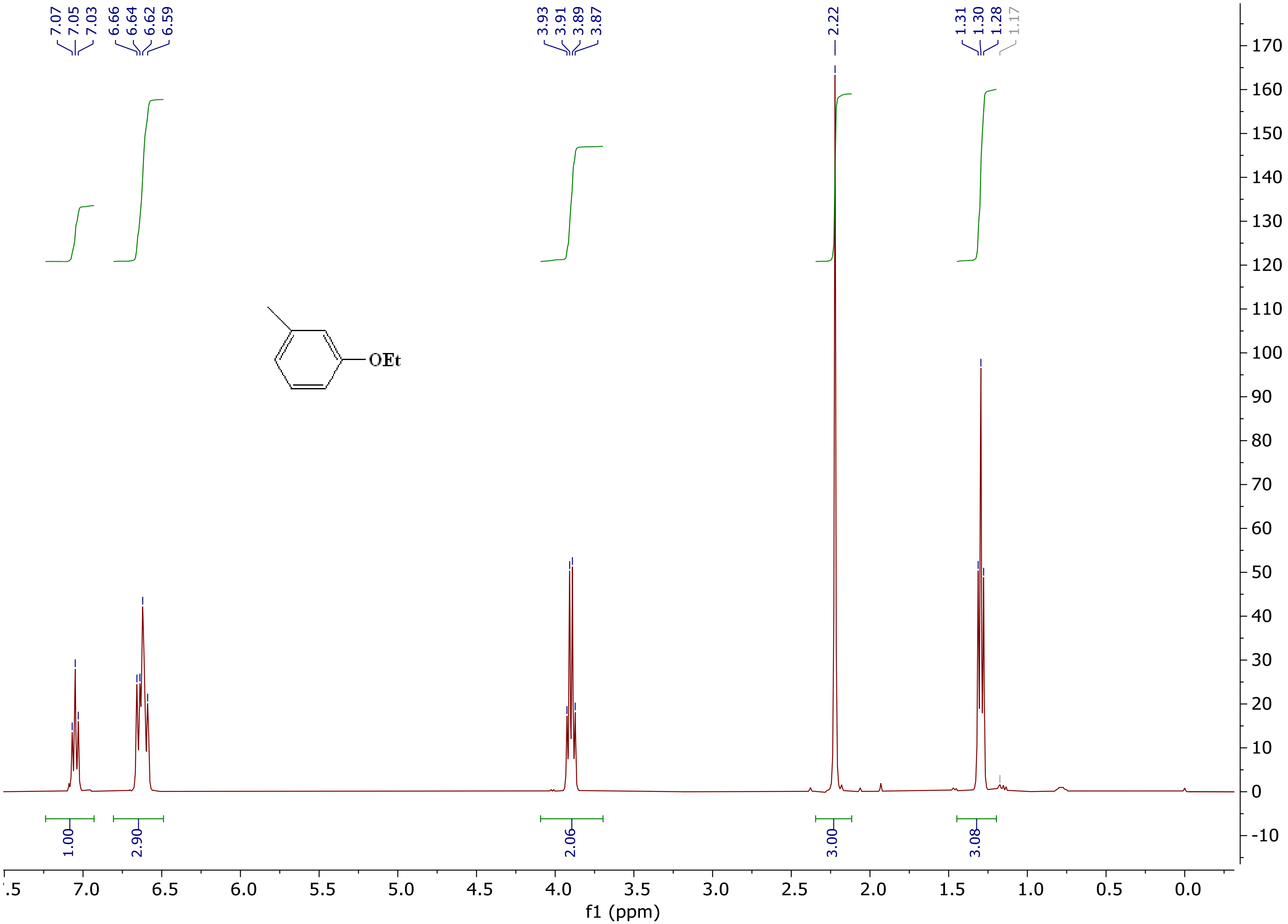

S17: ^{1}H-NMR spectrum of *m*-ethoxytoluene **9.**

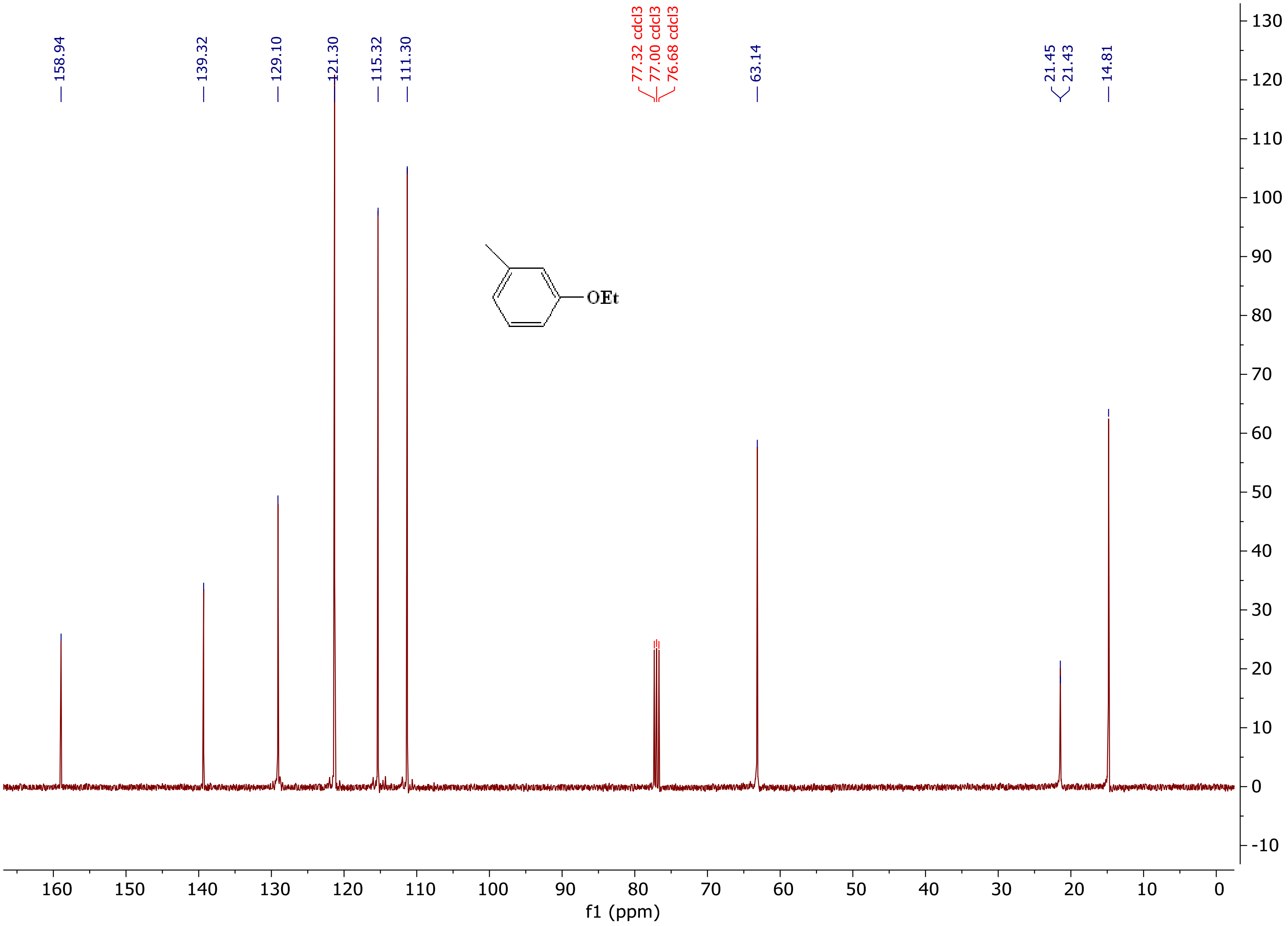

S18: ^{13}C-NMR spectrum of *m*-ethoxytoluene **9.**

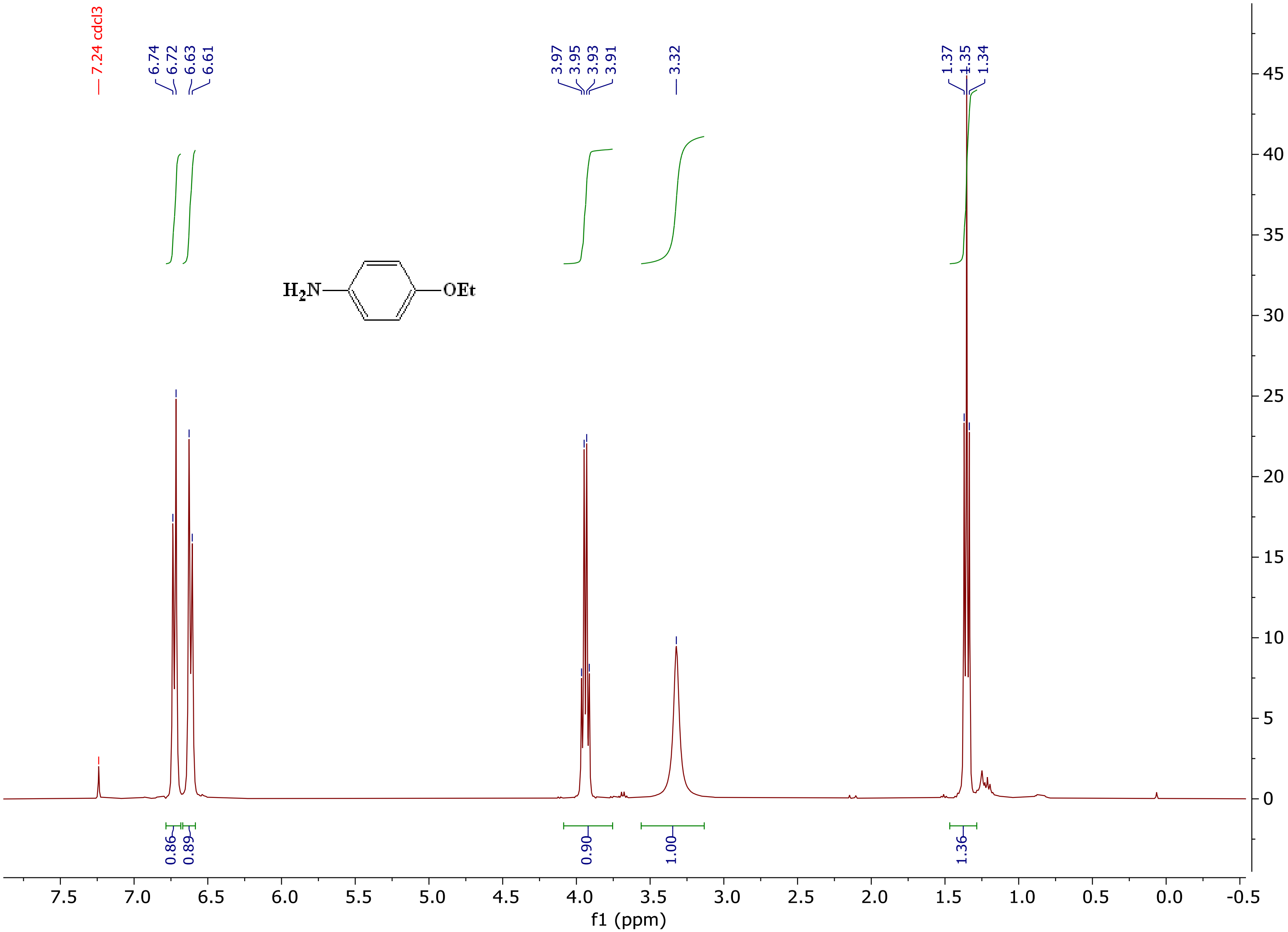

S19: ^{1}H-NMR spectrum of *p*-aminoethoxybenzene **10.**

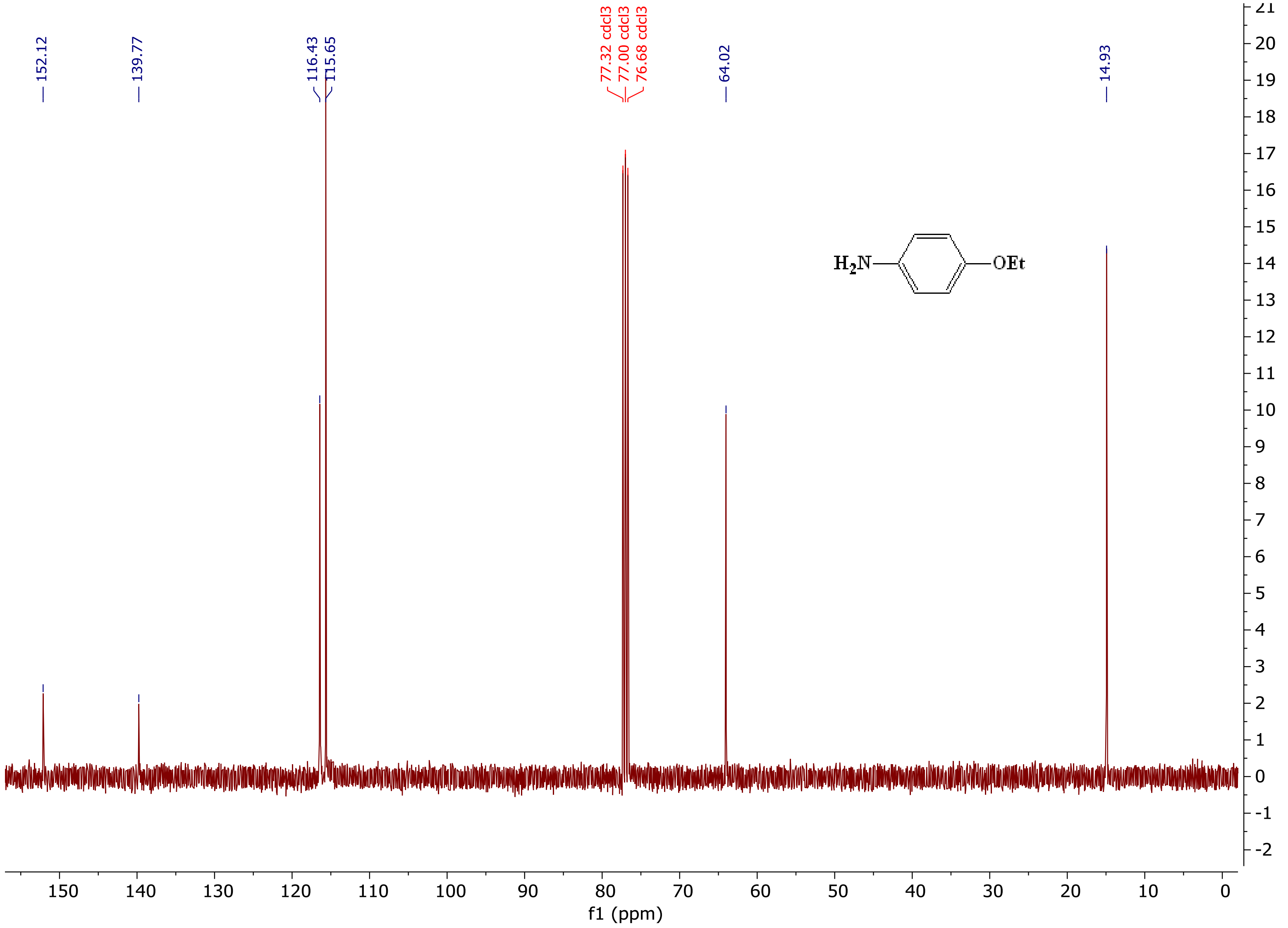

S20: ^{13}C-NMR spectrum of *p*-aminoethoxybenzene **10.**

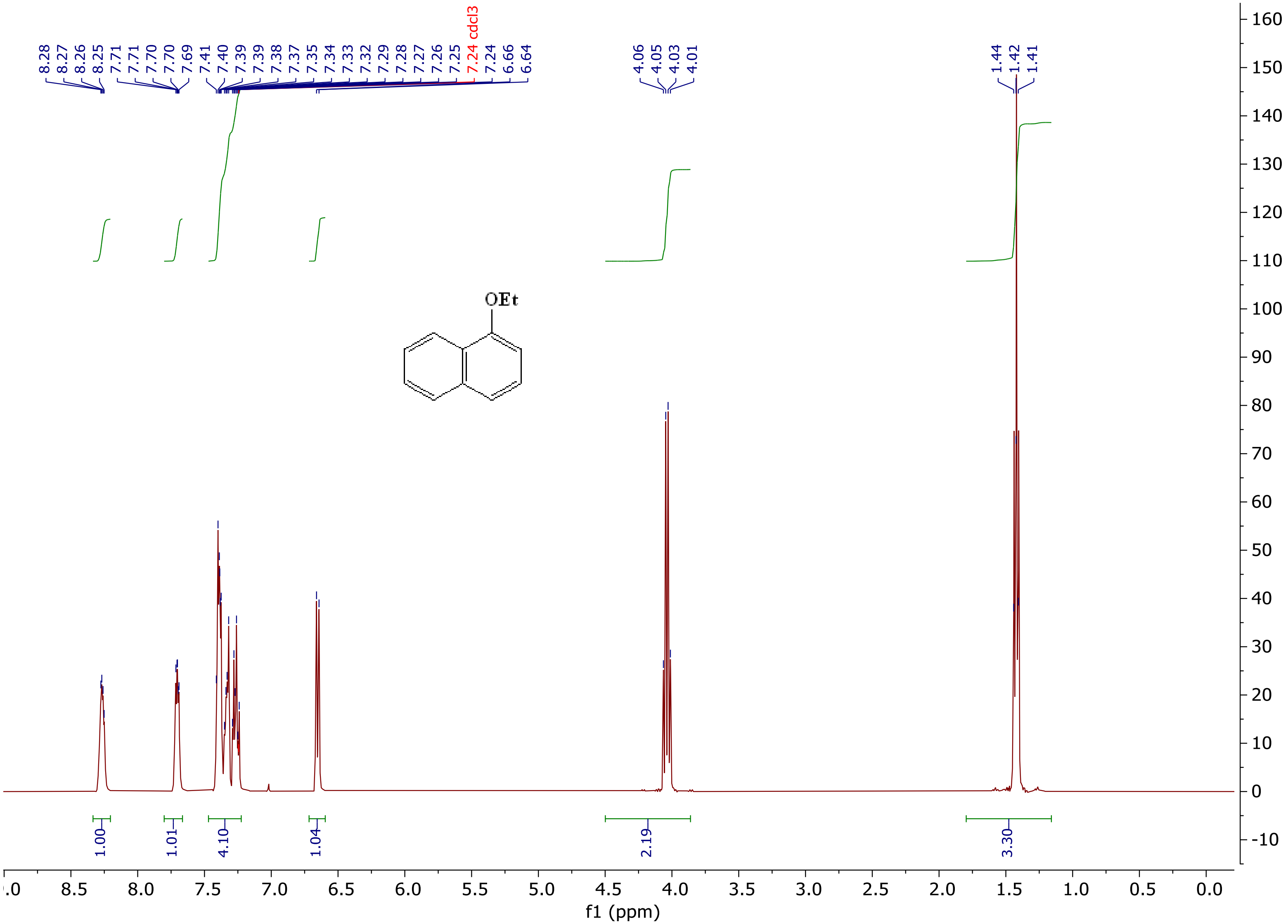

S21: ^{1}H-NMR spectrum of *α*-ethoxynaphthalene **11.**

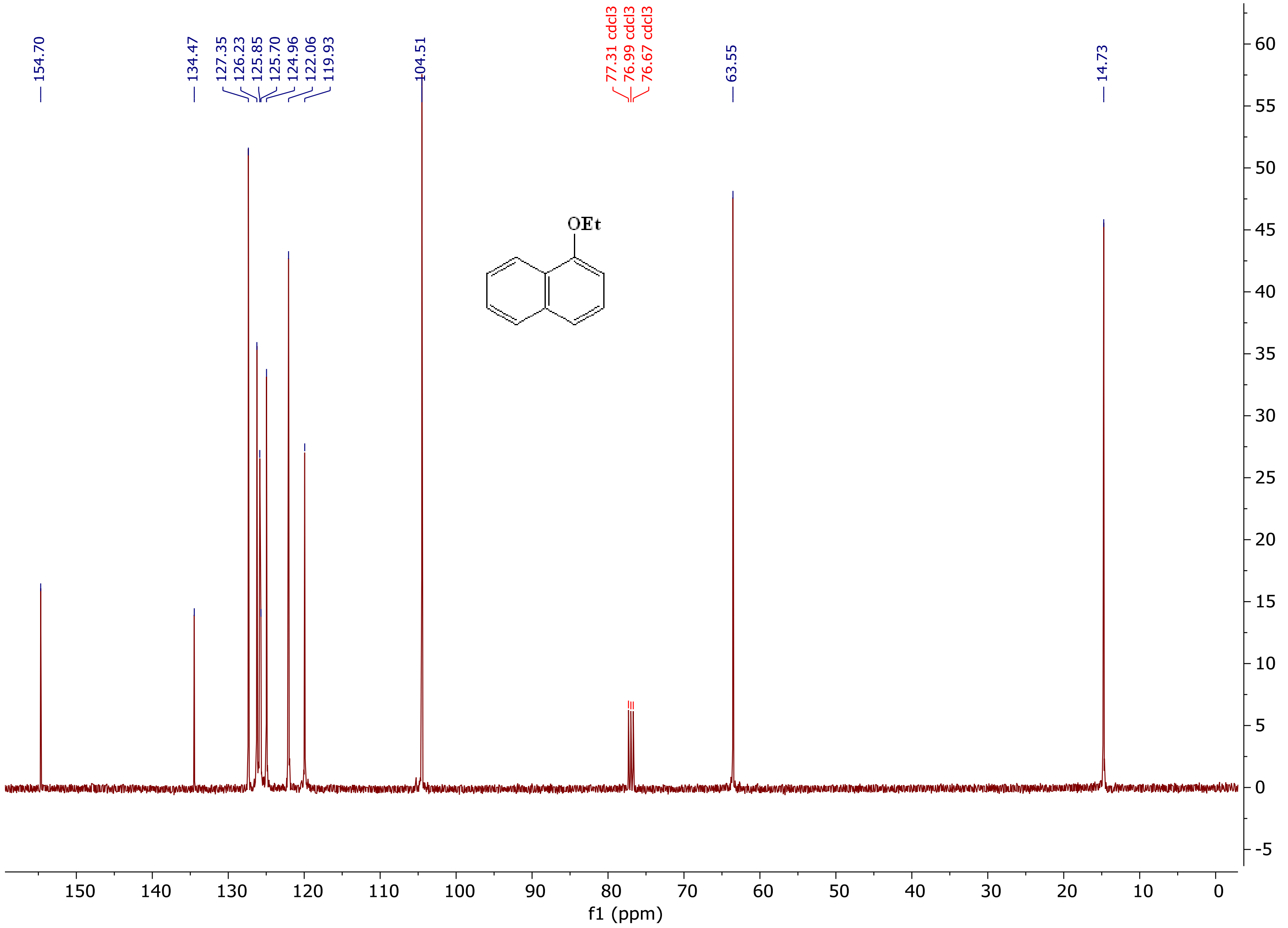

S22: ^{13}C-NMR spectrum of *α*-ethoxynaphthalene **11.**

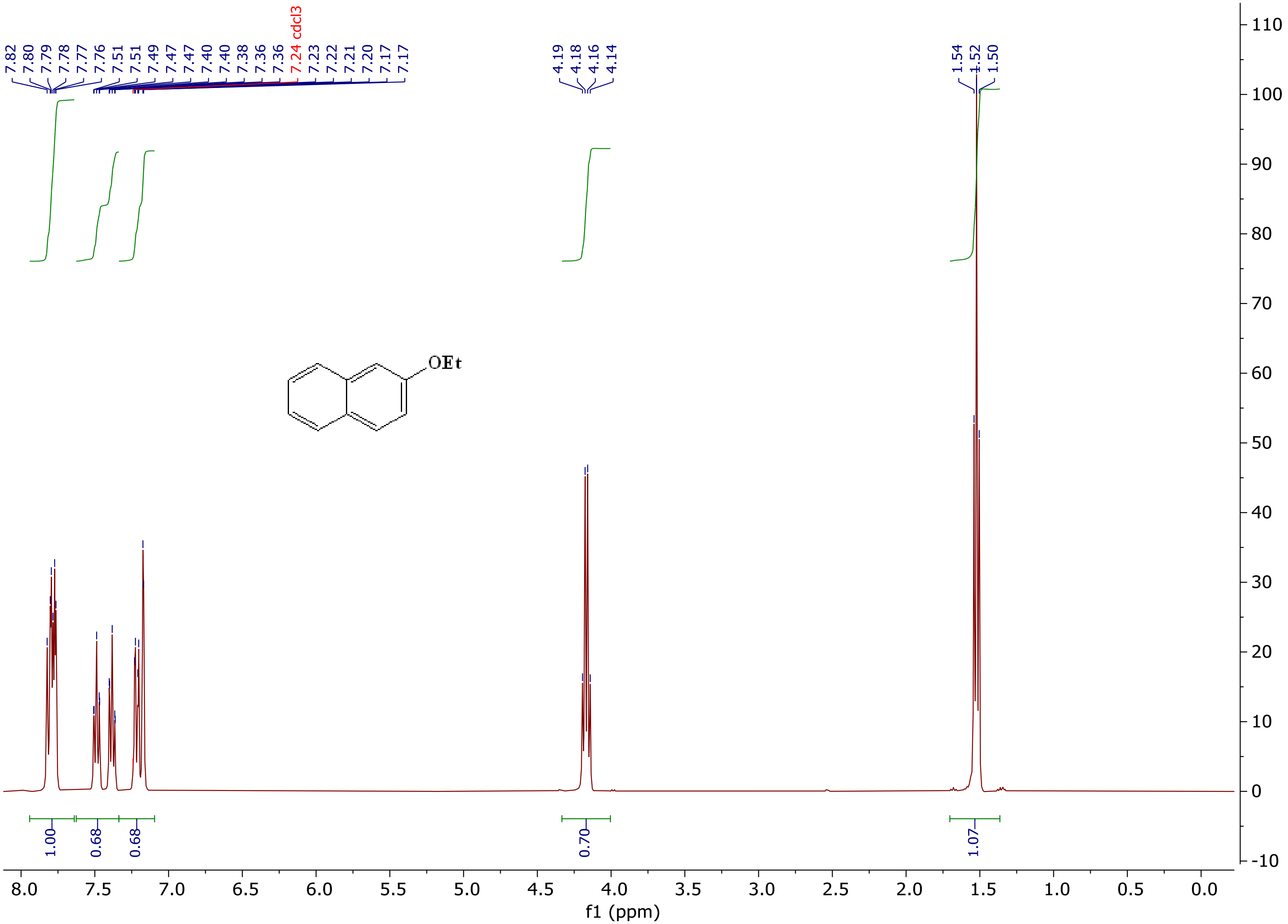

S23: ^{1}H-NMR spectrum of β-ethoxynaphthalene **12.**

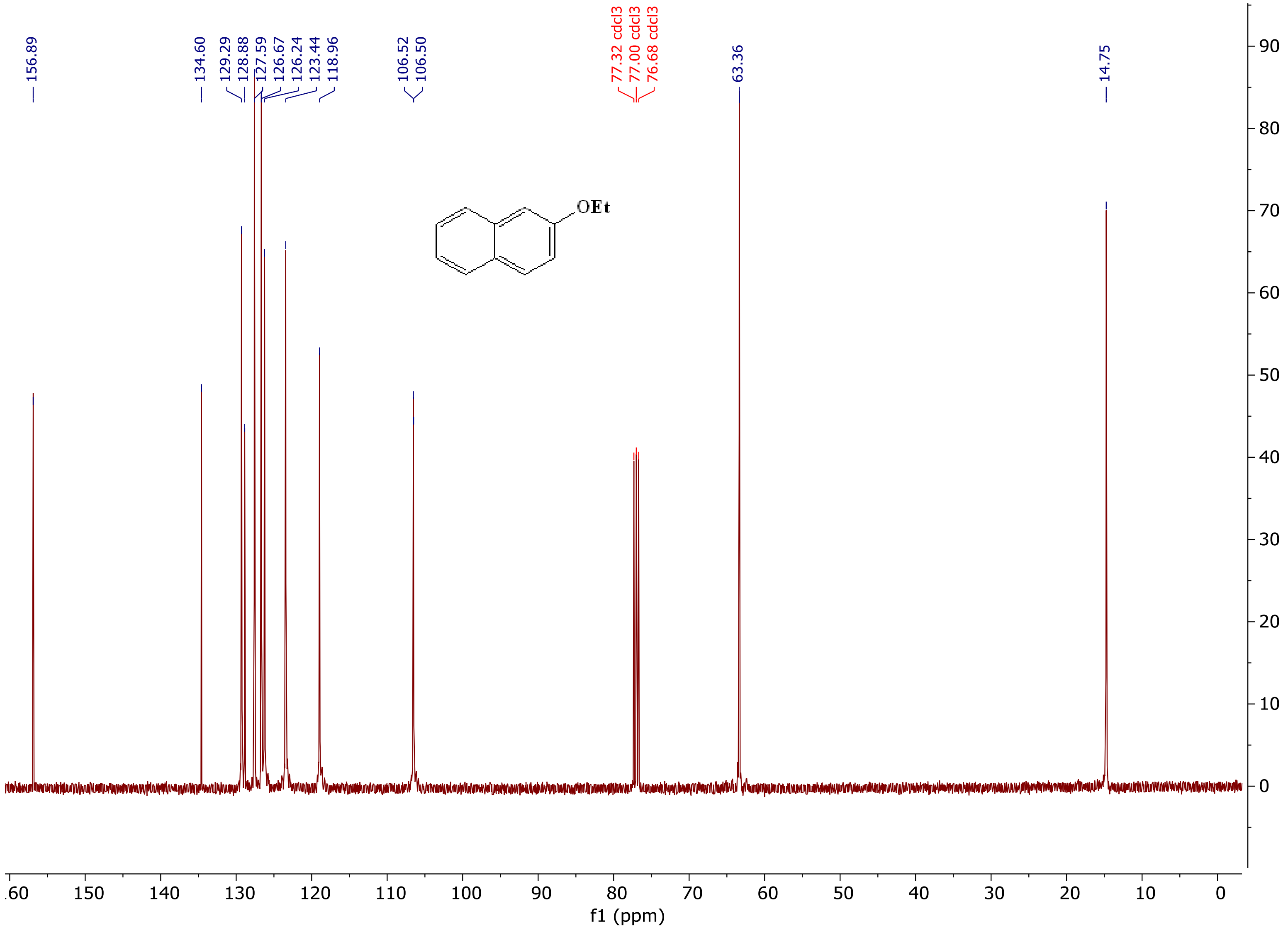

S24: ^{13}C-NMR spectrum of *β*-ethoxynaphthalene **12.**

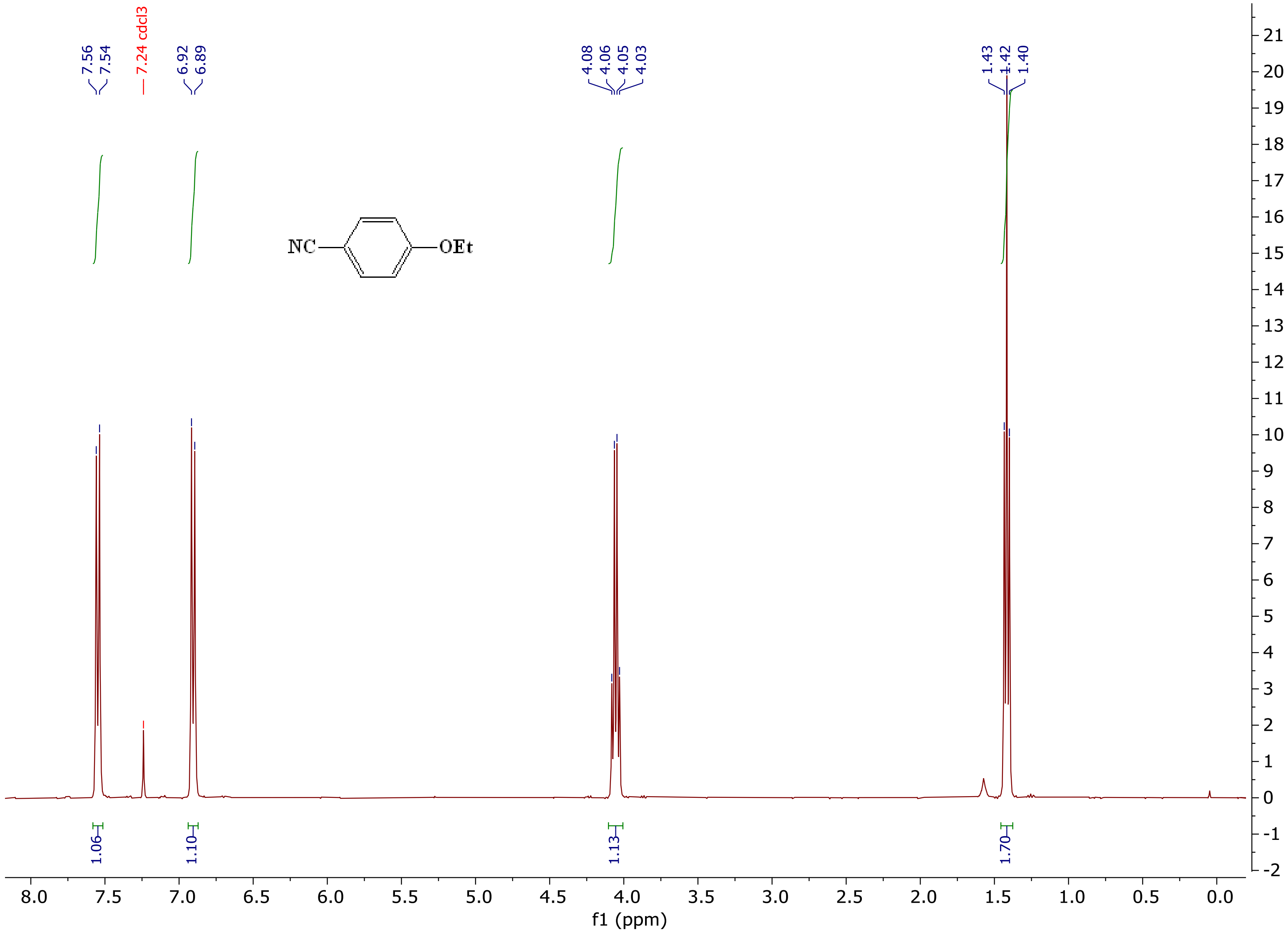

S25: ^{1}H-NMR spectrum of *p*-ethoxybenzonitrile **13.**

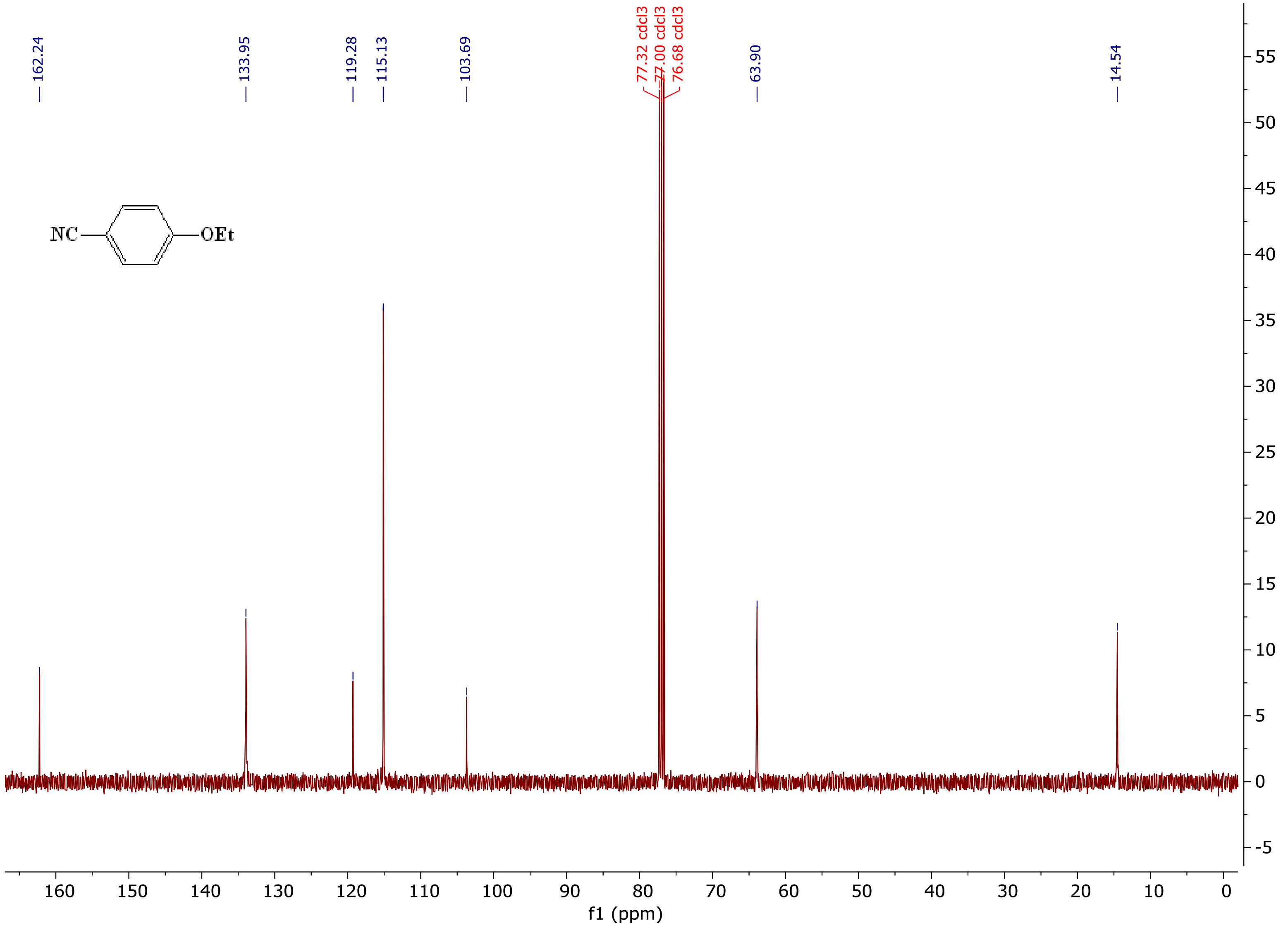

S26: ^{13}C-NMR spectrum of *p*-ethoxybenzonitrile **13.**

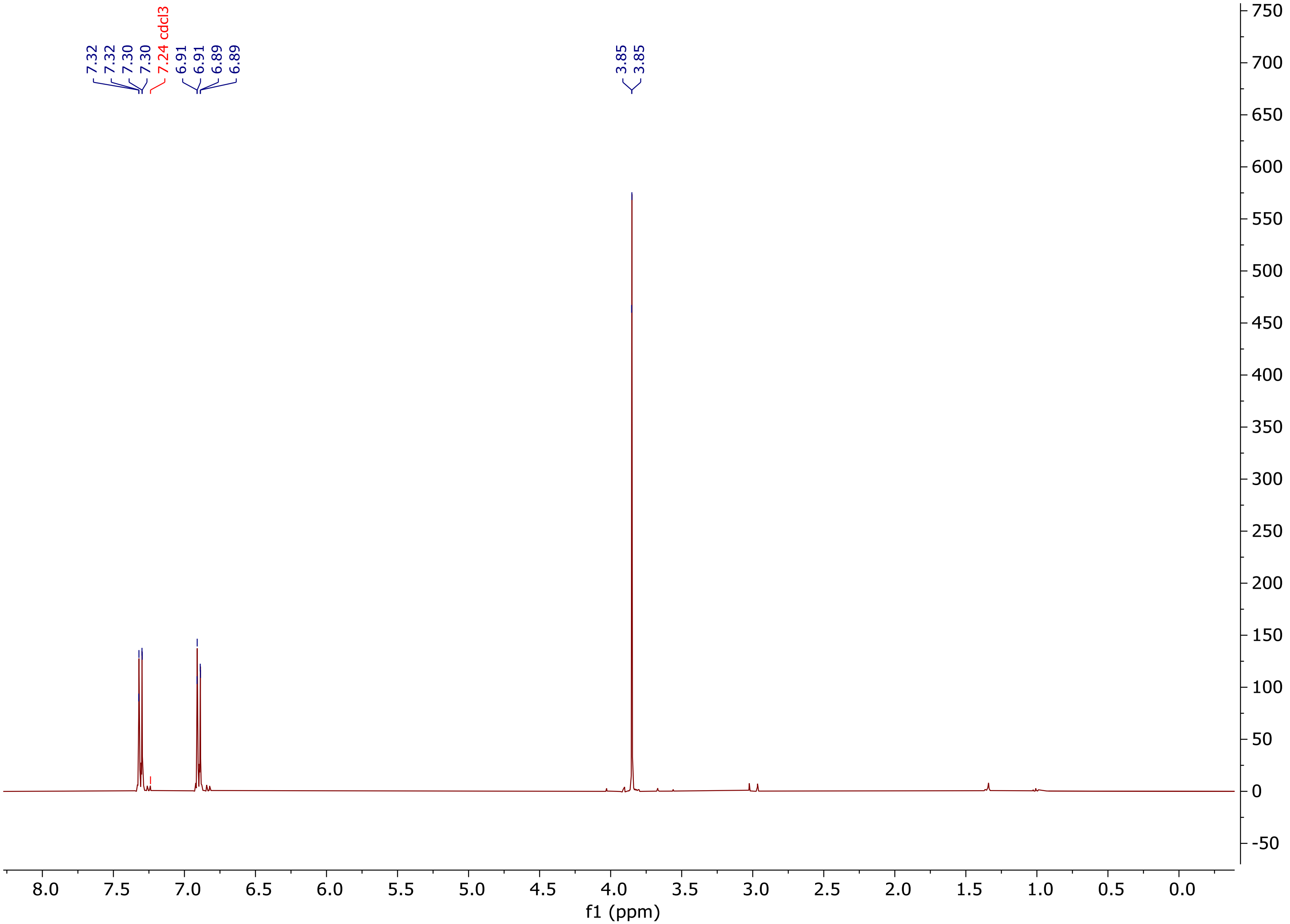

S27: ^{1}H-NMR spectrum of *p*-chloroanisole **14.**

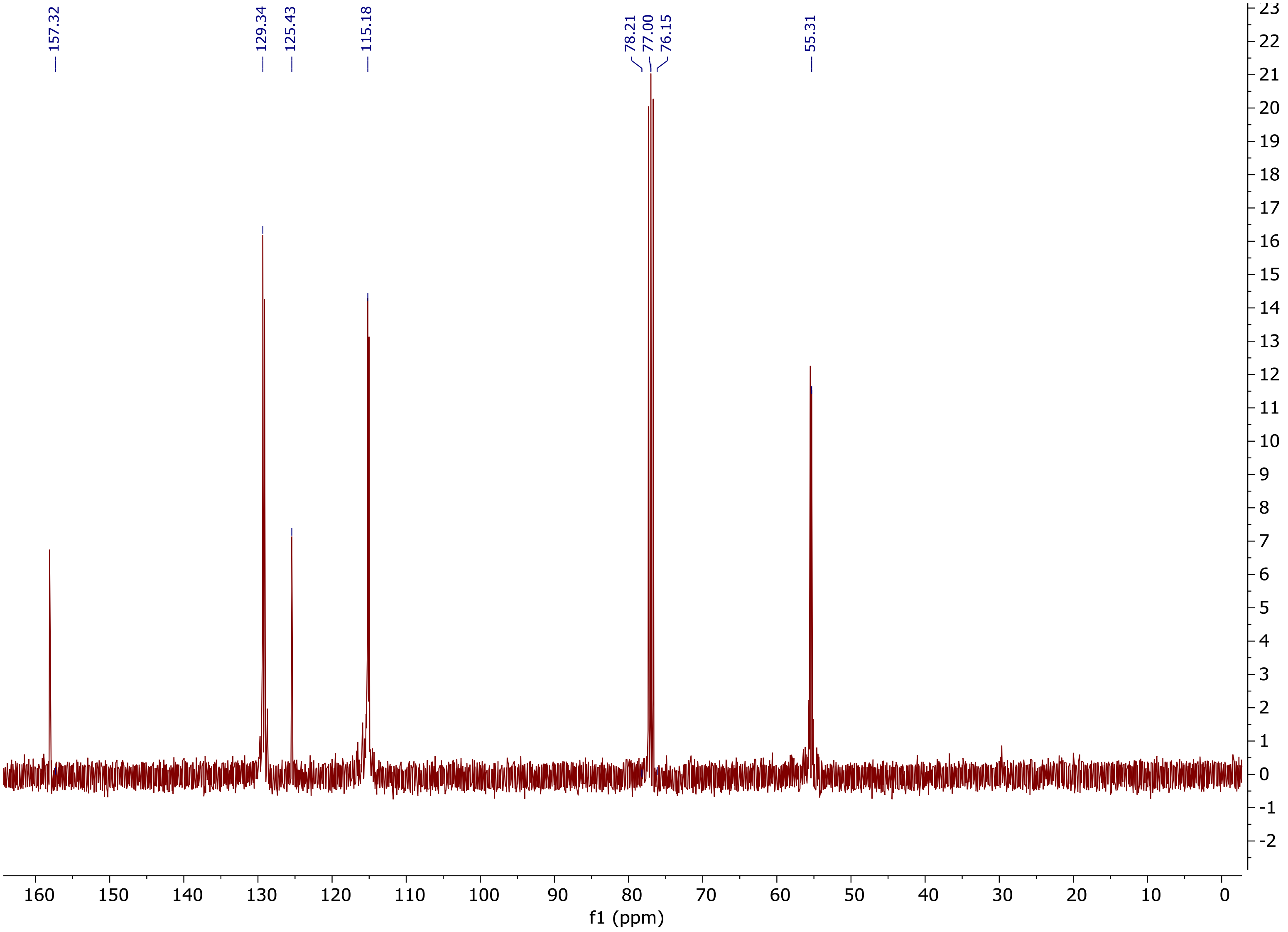

S28: ^{13}C-NMR spectrum of *p*-chloroanisole **14.**

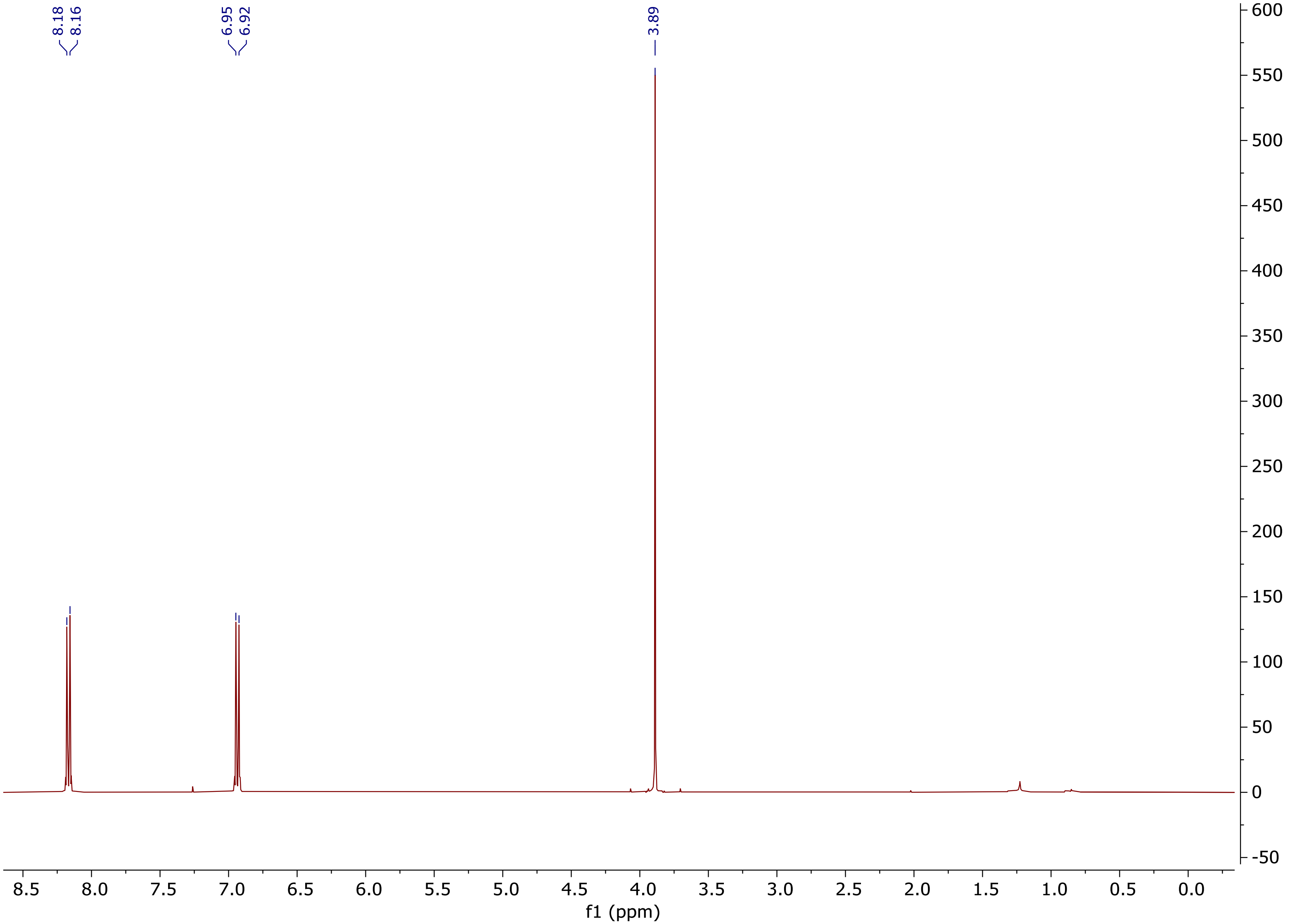

S29: ^{1}H-NMR spectrum of *p*-nitroanisole **15.**

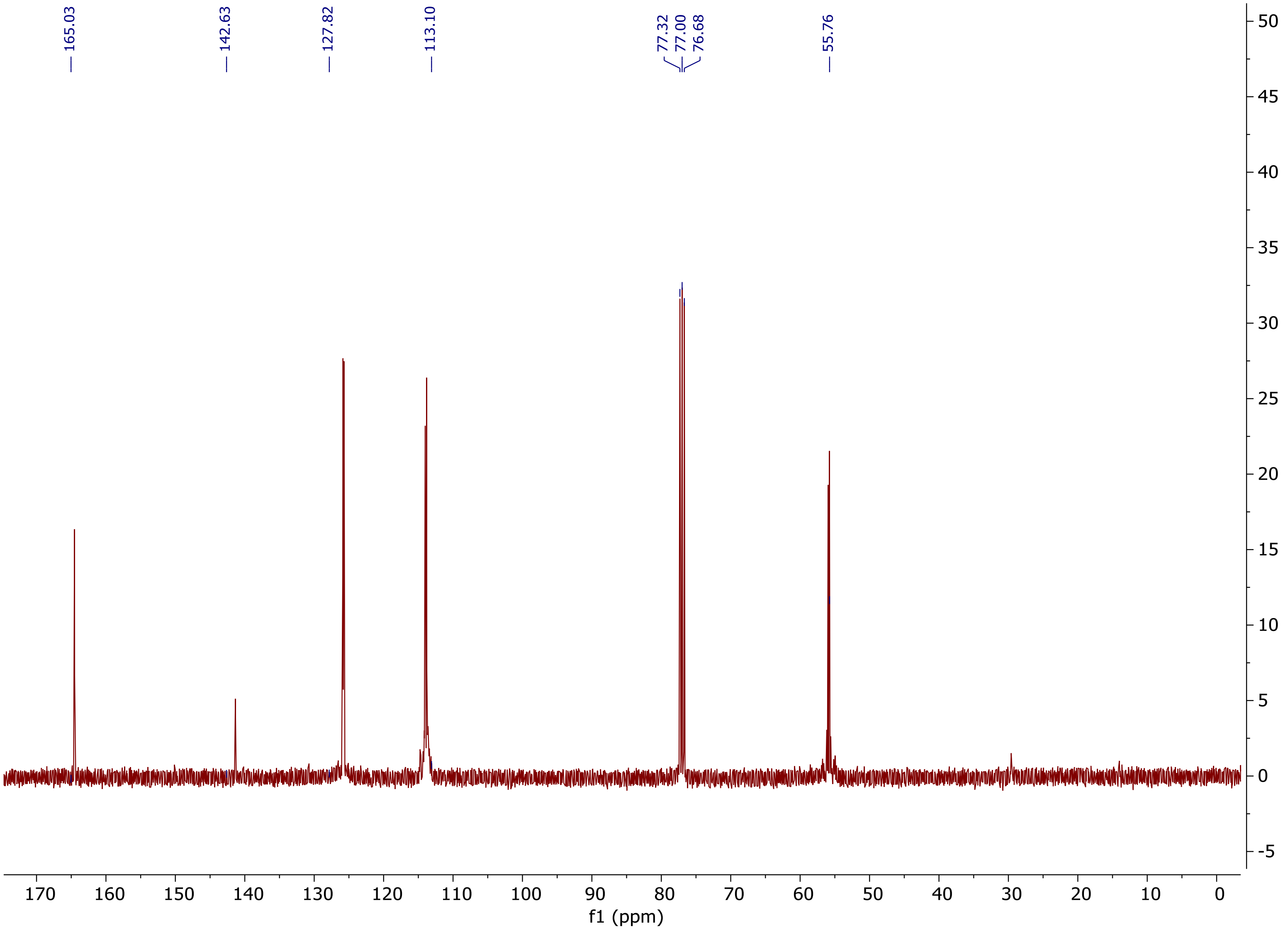

S30: ^{13}C-NMR spectrum of *p*-nitroanisole **15.**

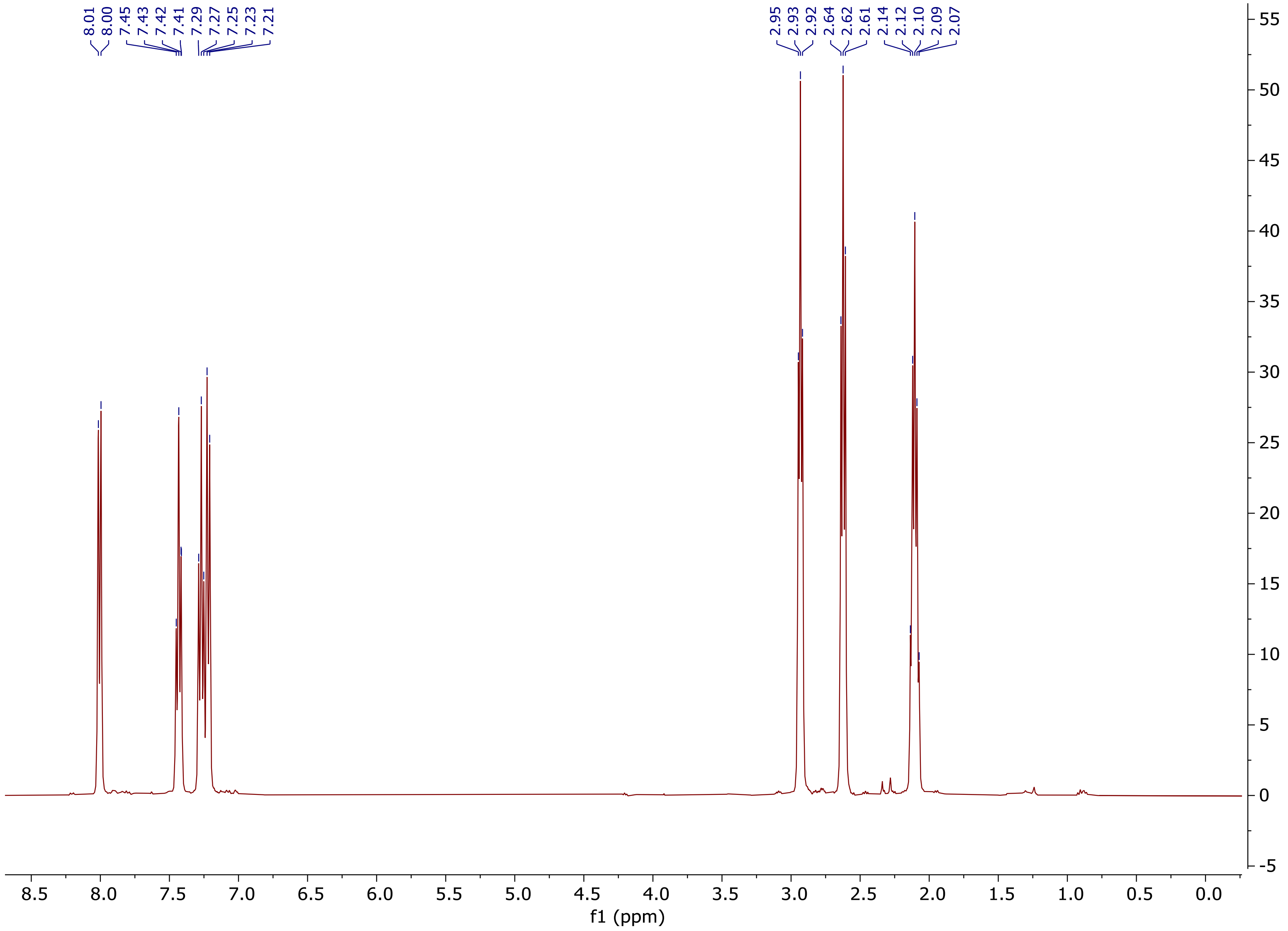

S31: ^{1}H-NMR spectrum of α-tetralone **16.**

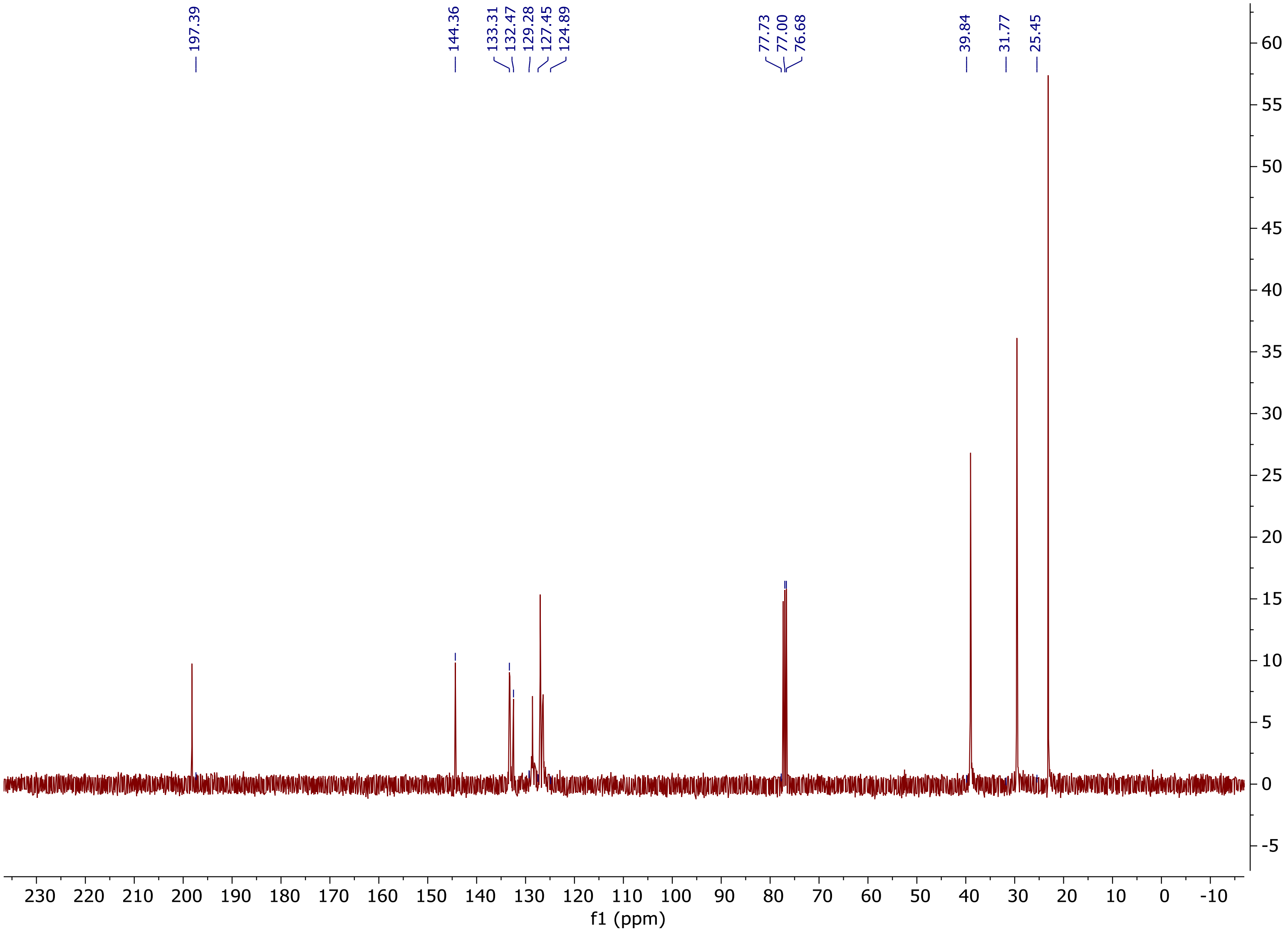

S32: ^{13}C-NMR spectrum of *α*-tetralone **16.**

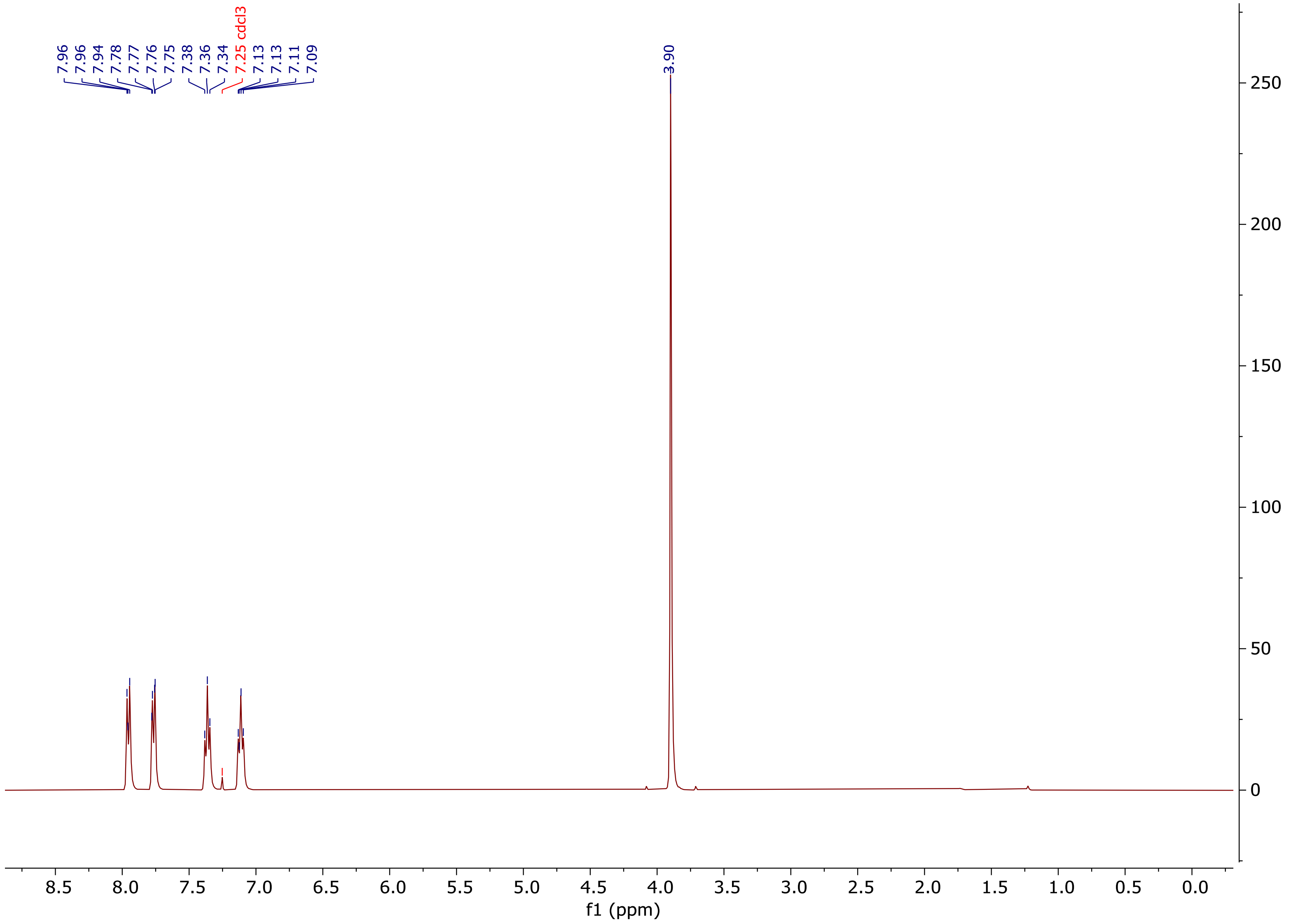

S33: ^{1}H-NMR spectrum of methyl *o*-iodobenzoate **17.**

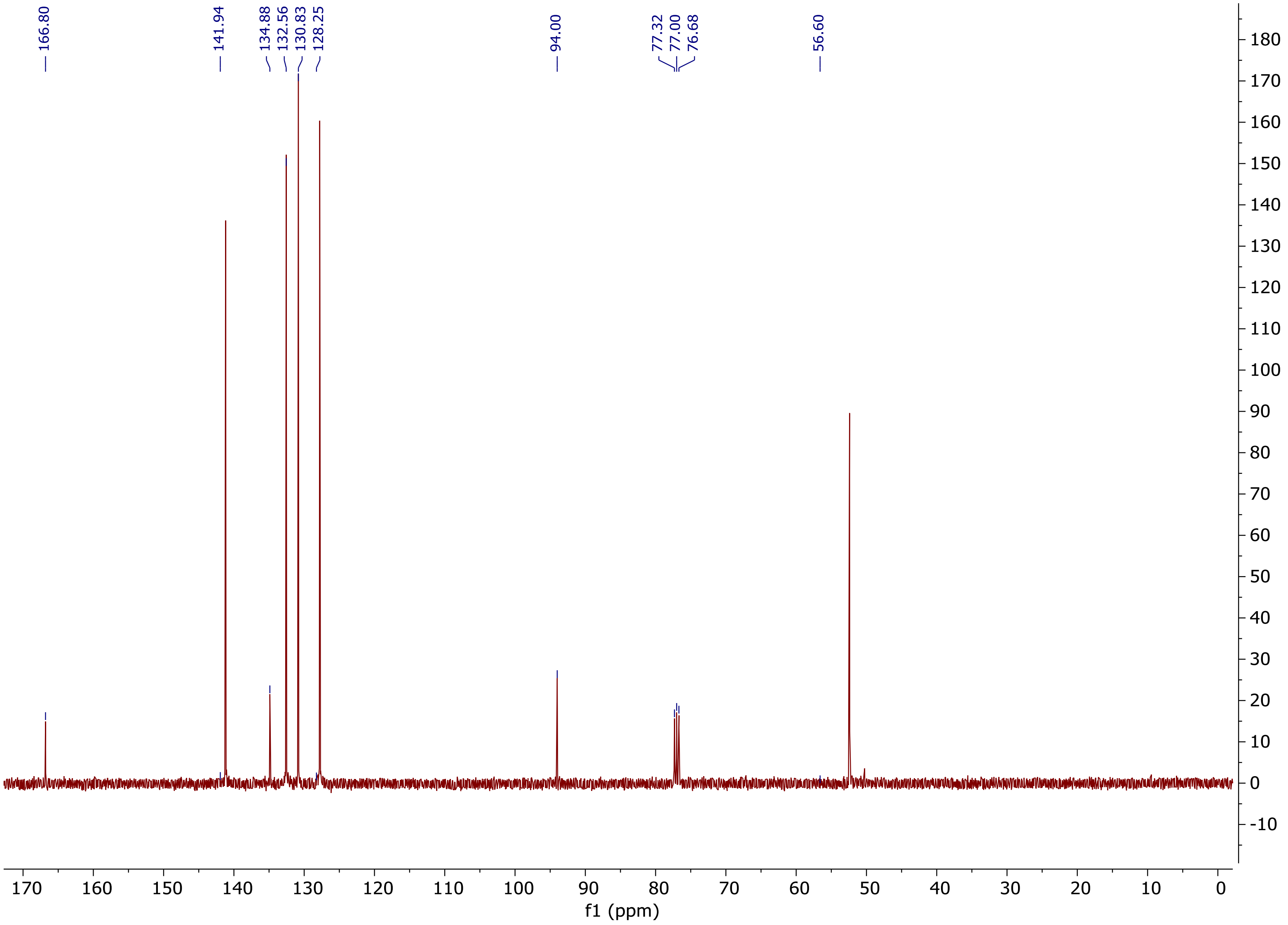

S34: ^{13}C-NMR spectrum of methyl *o*-iodobenzoate **17.**
